# Neuroimmune Regulation of Microvascular Inflammation: The Heart–Brain Axis, Mast Cells, and the Protective Role of Flavonoids—A Comprehensive Review

**DOI:** 10.3390/biomedicines14051073

**Published:** 2026-05-08

**Authors:** Paraskevi Papadopoulou, Theoharis C. Theoharides

**Affiliations:** 1 Department of Natural Sciences, Deree—The American College of Greece, 15342 Athens, Greece; vivipap@acg.edu; 2 Institute for Neuro-Immune Medicine, Nova Southeastern University, Clearwater, FL 33759, USA; 3 Department of Immunology, Tufts University School of Medicine, Boston, MA 02111, USA

**Keywords:** mast cells, microvascular inflammation, heart–brain axis, corticotropin-releasing hormone, neuroimmune regulation, flavonoids, therapeutic targets, vascular permeability, stress, cardiovascular disease

## Abstract

**Background/Objectives**: Cardiovascular disease (CVD), particularly coronary artery disease (CAD), is increasingly linked to microvascular inflammation driven by interactions between immune, vascular, and neuroendocrine systems. Mast cells (MCs), strategically positioned near blood vessels, play pivotal roles in this process through the release of inflammatory and vasoactive mediators, contributing to increased vascular permeability, endothelial dysfunction, and tissue inflammation in conditions including ischemia–reperfusion (I/R) and CVD. This comprehensive review examines the cellular and molecular mechanisms underlying MC-mediated microvascular inflammation, with emphasis on neuroimmune regulation through the heart–brain axis, and evaluates the therapeutic potential of flavonoids. **Methods**: A review of in vitro, animal, and clinical studies was conducted to assess MC-mediated cardiovascular pathology and the pharmacological effects of natural flavonoids on MC activation and microvascular inflammation. **Results**: Psychological and physical stress activates hypothalamic corticotropin-releasing hormone (CRH) signaling, directly triggering coronary MC degranulation via CRHR-1 and CRHR-2 receptors, while co-released neuropeptides, including neurotensin and urocortin, amplify this neuroimmune cascade. Traumatic brain injury, autonomic dysregulation, and atrial fibrillation further perpetuate this bidirectional heart–brain axis, linking neurological stress to microvascular injury and adverse cardiac remodeling. An autocrine–paracrine CRH amplification loop sustains chronic coronary microvascular inflammation, contributing to heart failure with preserved ejection fraction (HFpEF) and MC activation disease (MCAD)-related cardiovascular manifestations. Natural flavonoids were found to inhibit MC activation, suppress inflammatory mediator synthesis, and protect microvascular integrity through multiple molecular targets, including calcium signaling, transcription factors, oxidative stress pathways, and CRHR-1-mediated neuroimmune signaling. **Conclusions**: While challenges remain regarding bioavailability and standardization, multi-compound formulations targeting multiple risk factors hold promise for preventing CVD progression. Future research directions for advancing these natural compounds toward clinical implementation are identified.

## 1. Introduction

### 1.1. Background on Microvascular Inflammation

Microvascular inflammation represents a critical pathophysiological process underlying numerous disease states, characterized by the activation of immune cells, increased vascular permeability, and endothelial dysfunction [1,2,3]. The microcirculation, consisting of arterioles, capillaries, and venules, serves as the primary interface between circulating blood and tissue parenchyma, making it particularly susceptible to inflammatory insults [4]. Dysregulation of microvascular homeostasis contributes to the pathogenesis of CVD, metabolic disorders, neuroinflammatory conditions, and organ dysfunction following ischemia–reperfusion (I/R) injury [2,5].

The inflammatory response in the microcirculation involves complex interactions between resident immune cells, circulating leukocytes, and the vascular endothelium [6]. Among the various cellular mediators of microvascular inflammation, mast cells (MCs) have emerged as key orchestrators due to their strategic perivascular localization and rapid response capabilities [7]. These cells can initiate and amplify inflammatory cascades through the release of diverse bioactive mediators, ultimately leading to tissue damage and organ dysfunction [8]. Hence, MCs have been implicated in many diseases [7,9,10,11].

### 1.2. Mast Cells: Key Players in Microvascular Inflammation

Mast cells were first described by Paul Ehrlich in 1878 as granulated cells abundant in connective tissues [12,13]. Since then, extensive research has revealed their crucial roles in both physiological and pathological processes, particularly in immune surveillance and inflammatory responses [14,15,16]. These cells are strategically positioned throughout the body, with high concentrations near blood vessels, nerves, and mucosal surfaces, allowing them to serve as sentinels that can rapidly respond to tissue perturbations [14,17].

The unique characteristics of MCs include their ability to undergo rapid degranulation upon activation, releasing preformed mediators within seconds to minutes, followed by the synthesis and release of newly formed inflammatory compounds [13]. This biphasic response enables MCs to initiate immediate inflammatory reactions while sustaining and amplifying the inflammatory process through prolonged mediator release [18,19,20].

Recent advances in MC biology have revealed their involvement in diverse pathological conditions beyond classical allergic reactions, including CVD, neuroinflammation [21,22], and metabolic disorders [19]. The recognition of MCs as therapeutic targets has prompted investigations into novel approaches for modulating their activity and inflammatory responses [14,15,23].

### 1.3. Flavonoids as Natural Anti-Inflammatory Compounds

Flavonoids constitute a large family of polyphenolic compounds widely distributed in the plant kingdom, with over 6000 structurally diverse compounds identified to date [24]. These natural products are abundant in fruits, vegetables, tea, wine, and medicinal plants, representing one of the most consumed groups of phytochemicals in the human diet [25]. Flavonoids have a three-ring structure and can be subdivided based on oxy group, double bond, or hydroxyl group presence. They have antioxidant, anti-inflammatory, and antiproliferative properties, especially in inhibiting MC secretion [26].

Epidemiological studies have consistently associated higher flavonoid intake with reduced risk of chronic inflammatory diseases, CVDs, and certain cancers [26,27]. Flavonoids modulate reactive oxygen species (ROS)-scavenging enzymes, induce apoptosis and autophagy, and target key signaling pathways. The following review discusses the interconnected aspects of oxidative and inflammatory mechanisms, including lipid peroxidation, protein oxidation, DNA damage, and inflammation resolution. Epidemiological studies on flavonoids are also presented [28].

The therapeutic potential of flavonoids has attracted considerable scientific interest due to their diverse biological activities, including anti-inflammatory, antioxidant, antimicrobial, and immunomodulatory properties [29]. Specifically, their ability to modulate MC function and inflammatory responses has emerged as a promising therapeutic avenue for treating inflammatory and neurodegenerative disorders [30,31], including atherosclerosis [32].

The growing body of evidence supporting the anti-inflammatory effects of flavonoids, combined with their relatively favorable safety profiles, has positioned these compounds as attractive candidates for drug development and therapeutic intervention [26,33]. However, challenges related to bioavailability, standardization, and clinical translation remain significant barriers to their widespread therapeutic application [34].

This review integrates MC-driven microvascular inflammation with neuroimmune regulation along the heart–brain axis, highlighting corticotropin-releasing hormone-mediated signaling as a central mechanistic link between psychological stress and coronary pathology. In addition, it uniquely synthesizes emerging evidence on flavonoids as multi-target modulators of MC activation within this axis, providing a translational framework that connects molecular mechanisms to potential therapeutic strategies for cardiovascular disease.

### 1.4. Aims of the Review Paper

The primary aim of this comprehensive scoping review is to systematically examine and synthesize current knowledge regarding the role of MCs in microvascular inflammation and the therapeutic potential of flavonoids as natural anti-inflammatory agents targeting mast cell-mediated inflammatory responses.

Specific objectives include the following:

1. To comprehensively analyze the biological characteristics and microvascular localization of MCs, including their development, phenotypic diversity, and strategic positioning within the microvascular environment.
2. To elucidate the molecular mechanisms underlying MC-mediated microvascular inflammation, encompassing both preformed mediator release and de novo synthesis of inflammatory compounds, as well as the downstream effects on microvascular function.
3. To examine the pathophysiological roles of MCs across various inflammatory disease states, including ischemia–reperfusion injury, allergic and autoimmune disorders, CVD, and neuroinflammatory conditions.
4. To provide a detailed analysis of flavonoid structure, classification, and bioavailability, establishing the foundation for understanding their therapeutic potential.
5. To systematically review the anti-inflammatory effects of flavonoids on MCs, including mechanisms of degranulation inhibition, mediator synthesis suppression, and antioxidant protection.
6. To identify and analyze the molecular targets and signaling pathways through which flavonoids exert their beneficial effects on MC function and microvascular inflammation with emphasis on neuroimmune regulation through the heart–brain axis.
7. To critically evaluate experimental evidence and clinical studies supporting the therapeutic potential of flavonoids in treating MC-mediated inflammatory conditions.
8. To assess the current state of therapeutic potential and drug development involving flavonoids, including structure–activity optimization, formulation strategies, and regulatory considerations.
9. To identify current limitations, challenges, and future research directions in the field of flavonoid-based therapeutics for microvascular inflammation.
10. To provide evidence-based recommendations for future research priorities and clinical translation opportunities in this rapidly evolving field.

This review aims to serve as a comprehensive resource for biomedical students, researchers, clinicians, and pharmaceutical developers interested in understanding the intersection of MC biology, microvascular inflammation, and natural product therapeutics. By synthesizing current knowledge and identifying research gaps, this review will facilitate informed decision-making regarding future research directions and therapeutic development strategies.

## 2. Materials and Methods

This comprehensive/scoping review was conducted according to established methodological frameworks for reviews, following the guidelines outlined by Arksey and O’Malley and subsequently refined by Levac et al. and Mak & Thomas [35,36,37]. The specific review methodology was selected as the most appropriate approach to comprehensively map the existing literature on MCs, microvascular inflammation, and flavonoids, while identifying research gaps and opportunities for future investigation.

### 2.1. Search Strategy

A comprehensive search strategy was developed to identify relevant literature across multiple electronic databases. The search was conducted in PubMed/MEDLINE, Scopus, Web of Science, and Embase databases, covering publications from 2000 to March 2026. The search strategy combined controlled vocabulary terms (MeSH terms) and free-text keywords related to the key concepts.

Search terms included: mast cell* OR mastocyte*; microvascular OR microvessel* OR microcirculation; inflammation OR inflammatory; flavonoid* OR polyphenol* OR quercetin OR apigenin OR hesperidin OR fisetin OR luteolin OR nobiletin OR pygnogenol OR resveratrol; degranulation OR mediator release; and vascular permeability OR endothelial dysfunction.

Boolean operators (AND, OR) and truncation symbols were used to optimize search sensitivity and specificity. The search strategy was adapted for each database to account for differences in indexing and search functionalities.

### 2.2. Inclusion and Exclusion Criteria

Inclusion criteria: studies investigating MC biology and function; research examining microvascular inflammation mechanisms; studies evaluating flavonoid effects on MCs or inflammatory processes; both preclinical (in vitro and animal) and clinical studies; articles published in English; peer-reviewed journal articles, review articles, and conference abstracts; studies published from 2000 to March 2026.

Exclusion criteria: non-English language publications without English abstracts; case reports with fewer than 5 subjects; studies focusing exclusively on other cell types without MC relevance; articles not available in full text; duplicate publications.

### 2.3. Study Selection Process

The study selection process followed a two-stage screening approach.

Stage 1—Title and Abstract Screening: Two independent reviewers screened all retrieved titles and abstracts against the inclusion and exclusion criteria. Conflicts were resolved through discussion, and a third reviewer was consulted when consensus could not be reached.

Stage 2—Full-Text Screening: Full-text articles of potentially eligible studies identified in Stage 1 were independently assessed by three reviewers. The same conflict resolution process was applied. A total of 336 studies were included in this review.

**Figure 1:**
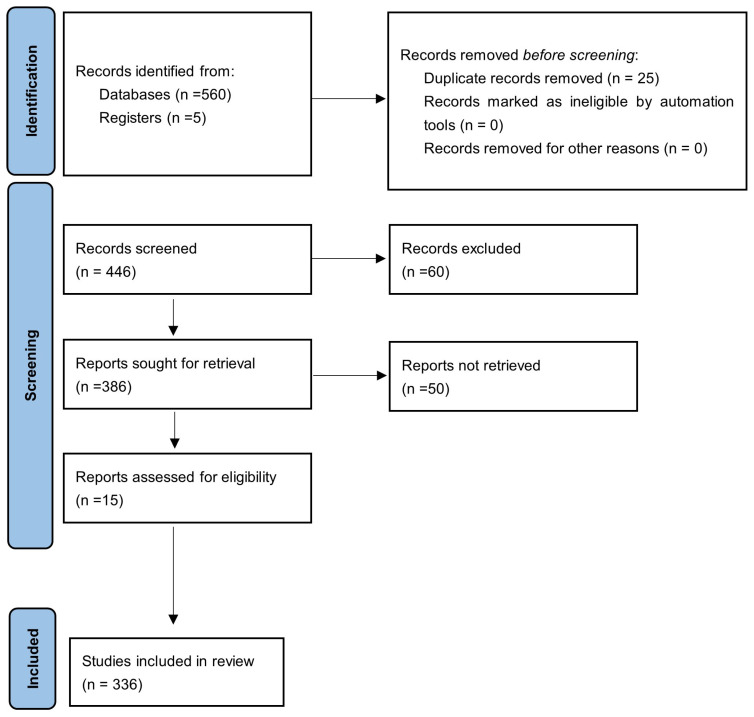
PRISMA 2020 flow diagram, which includes searches of databases, registers, and other sources.

The entire research model can be briefly described in Figure 1. The PRISMA 2020 flow diagram is used in systematic and scoping reviews, which include searches of databases, registers, and other sources [38].

### 2.4. Data Extraction and Synthesis

A standardized data extraction form was developed to capture relevant information from included studies. The following data were extracted: study characteristics (authors, year, study design, and sample size); population/model characteristics (species, cell lines, and disease models); intervention details (flavonoid compounds, concentrations, and administration methods); outcome measures (MC function, inflammatory markers, and clinical endpoints); key findings and conclusions; and study limitations and risk of bias considerations.

Data synthesis followed a narrative approach due to the heterogeneity of study designs, interventions, and outcome measures. Results were organized thematically according to the review objectives and presented in a structured format to facilitate comprehension and identification of knowledge gaps.

### 2.5. Limitations of the Methodology

Several limitations were acknowledged in this scoping review: language bias due to the inclusion of only English-language publications; publication bias favoring positive results; heterogeneity in study designs and outcome measures limiting quantitative synthesis; rapid evolution of the field requiring regular updates; and potential missed studies in the literature or non-indexed journals.

## 3. Results and Discussion

### 3.1. Mast Cell Biology and Microvascular Localization

#### 3.1.1. Mast Cell Development and Phenotypic Diversity

Mast cells originate from pluripotent hematopoietic stem cells in the bone marrow and circulate as committed progenitors before migrating to peripheral tissues, where they undergo final maturation [39,40]. The development of MCs is regulated by multiple transcription factors, including GATA-1, GATA-2, and MITF (microphthalmia-associated transcription factor), which control lineage commitment and phenotypic characteristics [41].

The stem cell factor (SCF) and its receptor c-Kit play essential roles in MC development, survival, and function [42]. SCF is constitutively produced by various cell types, including fibroblasts, endothelial cells, and epithelial cells, providing survival signals and promoting MC recruitment to specific tissue sites. The interaction between SCF and c-Kit also influences MC phenotype and functional characteristics [42]. In fact, SCF also stimulated MCs [43] via integrin β1 [44].

Mast cells exhibit remarkable phenotypic diversity depending on their tissue microenvironment and local cytokine milieu. Two major subtypes have been characterized in humans: tryptase-positive mast cells (MC_T_), predominantly found in the lungs and intestinal mucosa, and MCs containing both tryptase and chymase (MC_TC_), typically located in skin and other connective tissues. This phenotypic heterogeneity reflects functional specialization and adaptation to specific tissue requirements [45]. Recent evidence using RNA sequencing has identified additional subgroups of MCs in various tissues and pathologic conditions.

The maturation process involves the progressive accumulation of cytoplasmic granules containing preformed mediators, development of surface receptors, and acquisition of stimulus-response capabilities [46]. Table 1 is a summary of key findings on MC characteristics, phenotypic diversity, and strategic positioning in microvascular environments (Objectives 1 and 2).

#### 3.1.2. Strategic Positioning at Microvascular Sites

The perivascular distribution of MCs reflects their functional roles as immune sentinels and inflammatory mediators [57]. This strategic positioning enables MCs to monitor the vascular compartment [48] and initiate appropriate responses to pathological stimuli [58].

In the brain, MCs are found in close association with the blood–brain barrier (BBB), where they interact with astrocytes, pericytes, and endothelial cells as part of the neurovascular unit [59]. This anatomical relationship allows MCs to influence BBB permeability [60,61] and contribute to neuroinflammatory processes [62,63].

Mast cells are particularly involved in coronary dysfunction [3,55]. The interaction between MCs and the extracellular matrix plays a crucial role in their tissue localization and function. MCs express various integrins and adhesion molecules that facilitate their attachment to matrix components such as laminin, fibronectin, and vitronectin. These interactions not only anchor MCs in specific tissue locations but also influence their activation state and mediator release patterns [49].

The density and distribution of MCs in perivascular tissues can be altered in pathological conditions, with increased numbers often observed in inflammatory diseases, tumors, and tissue remodeling processes. This plasticity in MC distribution reflects their adaptive responses to changing tissue environments and pathological stimuli [64].

#### 3.1.3. Mast Cell Degranulation Mechanisms

Mast cell degranulation represents a highly regulated process involving the fusion of cytoplasmic granules with the plasma membrane, leading to exocytosis and the subsequent release of preformed mediators [65]. This process can be triggered through multiple pathways, with the classical IgE-mediated mechanism being the most extensively studied [66,67].

This pathway involves the cross-linking of IgE antibodies bound to the high-affinity IgE receptor (FcεRI) on the MC surface that initiates a complex signaling cascade involving calcium mobilization, protein tyrosine kinases, phospholipase C activation, and inositol trisphosphate generation. The resulting increase in intracellular calcium concentration triggers granule–plasma membrane fusion and mediator release [68].

Alternative activation pathways include complement-mediated degranulation through C3a and C5a receptors, neuropeptide-induced activation via substance P and vasoactive intestinal peptide receptors, and direct activation by certain drugs and physical stimuli [23]. These non-IgE-mediated pathways often involve different signaling mechanisms but ultimately converge on calcium-dependent exocytosis processes [69].

The degranulation process can be modulated by various factors, including the strength and duration of the stimulus, the presence of co-stimulatory signals, and the activation state of the MC. Selective release of specific mediators, has been observed under certain conditions, suggesting sophisticated regulatory mechanisms controlling mediator release [70,71].

### 3.2. Molecular Mechanisms of Mast Cell-Mediated Microvascular Inflammation

#### 3.2.1. Preformed Mediator Release

The cytoplasmic granules of MCs contain a diverse array of preformed mediators that can be rapidly released upon activation. Histamine, the most abundant and well-characterized granule constituent [72], exerts potent effects [73] on the microvasculature through H1 and H2 receptor activation. H1 receptor stimulation leads to endothelial cell contraction, increased vascular permeability, and vasodilation, while H2 receptor activation stimulates exocrine gland secretion, and can have both pro- and anti-inflammatory effects depending on the cellular context [7]. However, the effect of histamine on the cardiovascular system is complicated [74,75] and heterogeneous [76]. Histamine is primarily vasoconstrictive on the coronary vasculature [77,78,79] via reduction in cAMP [80]. However, histamine may be vasodilatory in proximal coronary vessels via increase in nitric oxide (NO) mediated via H2-receptor mediated cAMP increase [81,82] permitting leukocyte accumulation and inflammation, but vasoconstrictive in distal coronary vessels leading to anoxia and angina pain [83,84]. Granulocyte macrophage-colony stimulating factor (GM-CSF) can increase the expression of histamine and histamine receptors in monocytes/macrophages in areas of atherosclerosis [85]. These disparate actions appear to be mediated via activation of both H1 and H2 receptors [86,87,88,89,90]. In fact, platelet activating factor (PAF) was shown to increase synthesis of H1 receptors, thus contributing to the progression of CVD [91]. Histamine also stimulates the production of eicosanoid metabolites in human coronary endothelial cells [92,93].

Expression of thrombin receptors in human atherosclerotic coronary arteries leads to an exaggerated vasoconstrictive response [94] possibly via activation of MCs [95]. These actions have led to the concept of “allergic angina” [95] that has been known as “Kounis syndrome” [77,79].

Proteases represent another major class of preformed mediators [96], with tryptase and chymase being the most abundant [97]. Tryptase, a serine protease stored in all human MCs, can activate protease-activated receptors (PARs) on endothelial cells, leading to increased vascular permeability and inflammatory cell recruitment [98]. However, serum tryptase is elevated also in systemic mastocytosis and MC activation disorders, but also in hereditary-alpha-tryptasemia (HαT) chronic kidney disease and clonal myeloid disorders [99,100]. Chymase, present in MC_TC_-type MCs, can cleave a number of substrates [101] and generate angiotensin II from angiotensin I, contribute to tissue remodeling through matrix metalloproteinase (MMP) activation, and promote inflammatory responses [102,103]. In fact, chymase inhibitors have been discussed for the treatment of CVS diseases [104].

Heparin, a highly sulfated glycosaminoglycan, serves multiple functions beyond its anticoagulant properties. It can modulate complement activation, interact with chemokines and growth factors, and influence angiogenesis and tissue repair processes. Other granule constituents include carboxypeptidase A, cathepsin G, and various cytokines such as TNF-α and IL-4 that are prestored in granules [105].

The kinetics of preformed mediator release enables MCs to initiate immediate inflammatory responses within seconds to minutes of activation. This rapid response capability is particularly important in the context of microvascular inflammation, where prompt changes in vascular permeability and blood flow can have significant physiological consequences [7].

#### 3.2.2. De Novo Synthesis and Release of Inflammatory Mediators

Following degranulation, activated MCs initiate the synthesis and release of newly formed inflammatory mediators, including lipid mediators, cytokines, and chemokines. The arachidonic acid cascade is rapidly activated, leading to the production of prostaglandins, leukotrienes, and other bioactive lipids. Prostaglandin D2 (PGD2), the major cyclooxygenase product in MCs, promotes vasoconstriction, sensory nerve sensitization, bronchoconstriction, and inflammatory cell recruitment, especially TH2 cells bronchoconstriction, and inflammatory cell recruitment [106].

Leukotriene synthesis through the 5-lipoxygenase pathway generates potent inflammatory mediators including LTC4, LTD4, and LTE4. These cysteinyl leukotrienes increase vascular permeability, promote smooth muscle contraction, and enhance inflammatory cell recruitment and activation. The production of PAF [107] by activated MCs contributes to platelet aggregation, enhanced vascular permeability, and amplification of inflammatory responses [108]. Interestingly, use of MC ‘stabilizers’ have been considered as treatment of Kounis syndrome [109,110]. Platelets can engage MCs in a bilateral IL-33 driven loop [111].

Cytokine production by MCs involves the activation of multiple transcription factors, including NF-κB, AP-1, and NFAT. TNF-α represents one of the most important MC-derived cytokines, capable of promoting endothelial activation, leukocyte adhesion, and tissue inflammation [112]. Other significant cytokines include IL-1β, IL-6, IL-13, IL-31, IL-33 [113,114] and various chemokines such as CCL2 (MCP-1) and CXCL8 (IL-8) that promote inflammatory cell recruitment. This prolonged mediator release phase is crucial for the development of chronic inflammatory conditions and tissue remodeling processes [115]. FcεRI signaling influences exosome composition and function [116]. MC mediators, such as TGF beta can contribute to cardiac fibrosis [117].

#### 3.2.3. Signaling Pathways in Mast Cell Activation

The activation of MCs involves complex intracellular signaling networks that coordinate degranulation, mediator synthesis, and cellular responses. The FcεRI-mediated signaling pathway has been extensively characterized and serves as a prototype for understanding MC activation mechanisms [68,118,119]. The FcεRI IgE found on MCs and basophils, is phosphorylated by Src family kinase Lyn and activated by tyrosine kinase Syk. Researchers found two non-canonical Syk binding modes and a novel role of the Src homology 2 domain-containing inositol 5′-phosphatase 1 (SHIP1) in regulating Syk activity. These findings suggest that the signalosome’s composition and dynamics influence immunoreceptor signaling activities [120]. Upon FcεRI cross-linking, the receptor-associated Src family kinases Lyn and Fyn become activated, leading to phosphorylation of immunoreceptor tyrosine-based activation motifs (ITAMs) in the receptor subunits. This initiates a signaling cascade involving Syk kinase activation, phospholipase C-γ (PLCγ) activation, and the generation of second messengers inositol 1,4,5-trisphosphate (IP3) and diacylglycerol (DAG) [120]. The increase in intracellular calcium concentration, resulting from IP3-mediated calcium release from intracellular stores and calcium influx through store-operated channels, is essential for degranulation [120]. Calcium-dependent protein kinase C (PKC) isoforms are activated by DAG and contribute to both degranulation and cytokine gene transcription [120].

The mitogen-activated protein kinase (MAPK) pathways, including ERK1/2, p38, and JNK, are activated downstream of FcεRI and contribute to cytokine production and cellular responses [121]. These pathways regulate the activation of transcription factors such as NF-κB, AP-1, and NFAT that control the expression of inflammatory genes.

The PI3K/Akt pathway plays important roles in MC survival, degranulation, and cytokine production. Recent studies have highlighted the involvement of the mammalian target of rapamycin (mTOR) pathway in regulating MC function, particularly in the context of metabolic regulation and autophagy [122]. The dysregulated PI3K/Akt/mTOR signaling pathway is linked to immune-mediated inflammatory and hyperproliferative dermatoses. Inhibitors of mTOR activation, such as rapamycin, one in MC but are limited by severe side effects [122]. Natural products and synthetic compounds are being considered for inhibition of this pathway [123].

#### 3.2.4. Microvascular Response to Mast Cell Mediators

The release of MC mediators triggers complex responses in the microvascular endothelium that collectively contribute to the inflammatory process. Histamine-induced endothelial cell contraction results in the formation of intercellular gaps, leading to increased vascular permeability and plasma protein extravasation. This process is mediated through H1 receptor activation, calcium mobilization, and cytoskeletal reorganization [124].

Mast cell-derived TNF-α promotes endothelial activation through NF-κB-dependent upregulation of adhesion molecules (ICAM-1, VCAM-1, E-selectin) and chemokines. This endothelial activation facilitates leukocyte adhesion and transmigration, amplifying the inflammatory response. The production of nitric oxide by activated endothelial cells contributes to vasodilation and can have both pro- and anti-inflammatory effects [125].

Coagulation factors, responsible for blood clot formation, can activate PARs in vascular smooth muscle cells (VSMCs). In addition, MCs synthesize and secrete thromboxane and PAF that contribute to clot formation [107]. During vascular repair, VSMCs switch to a reparative phenotype, but prolonged stress leads to vascular remodeling, contributing to cardiovascular disease. Understanding PAR activation mechanisms is crucial for developing novel therapeutic strategies to address vascular remodeling and improve vessel wall integrity [103].

Furthermore, the complement is an essential immune system that defends against pathogens and maintains host homeostasis. It is activated by conformational changes in recognition molecular complexes, regulated to avoid tissue damage. Recent advances in understanding the molecular and structural basis of complement pathways, their implications on physiology and pathology, and the development of complement inhibitor therapies [126]. The complement system can be activated by MC-derived mediators, leading to the generation of anaphylatoxins C3a and C5a that further promote inflammation [126]. This creates positive feedback loops that amplify and sustain the inflammatory response. The interaction between MC mediators and the coagulation system can also contribute to thrombotic complications in inflammatory conditions. Table 2 shows the key mechanisms of MC activation, mediator release, and downstream effects on microvascular function (Objectives 2 and 3).

### 3.3. Pathophysiological Roles in Disease States

#### 3.3.1. Allergic and Autoimmune Disorders

Mast cells are central mediators of allergic reactions, from localized cutaneous responses to life-threatening systemic anaphylaxis [16]. The IgE-mediated activation of MCs in sensitized individuals leads to rapid mediator release and the characteristic symptoms of allergic reactions. The microvascular effects of MC activation, including increased permeability and vasodilation, contribute to tissue swelling, erythema, and hypotension [56].

Anaphylaxis is a severe allergic reaction triggered by immune activation and mediator release. Research has primarily focused on the immune component, but new approaches are needed to understand the involvement of other cellular and molecular agents. The vascular niche receives signals like histamine, which trigger anaphylactic events. Cardiovascular manifestations, such as increased vascular permeability and vasodilation, are crucial in the pathophysiology of anaphylaxis. Understanding vascular physiology and its molecular mechanisms can improve clinical management and treatment options for anaphylaxis [56].

In chronic allergic conditions such as asthma, chronic urticaria, and atopic dermatitis, persistent MC activation contributes to ongoing inflammation, tissue remodeling, and organ dysfunction. The prolonged release of inflammatory mediators promotes fibroblast proliferation, collagen deposition, and structural changes that characterize these chronic conditions [137]. Advancements in understanding type 2 immunity in allergic diseases like asthma, rhinitis, and atopic dermatitis have led to advancements in understanding its mechanisms. Type 2 immunity protects against parasitic diseases, expels parasites, maintains epithelial barriers, and counterbalances the type 1 immune response. It originates from epithelial cells and innate lymphoid cells, resulting in adaptive T and B-cell immunity, particularly IgE antibody production. Recent biologics targeting IL4/IL13, IL-5, and IgE have shown promising results for almost all ages, but some patients with severe allergic diseases still do not respond [137].

It is now well recognized that patients with MC activation disorders (MCADs), especially MC activation syndrome (MCAS), could have symptoms consistent with MCAD [138,139,140,141]. A case in point is coronary hypersensitivity or Kounis syndrome [142,143].

The interaction between MCs and adaptive immune responses involves both direct effects on T and B cells and indirect effects through antigen-presenting cell activation [144,145,146]. Mast cell-derived mediators can influence T cell polarization, promoting Th2 responses in allergic conditions and potentially contributing to autoimmune pathology [147].

#### 3.3.2. Ischemia–Reperfusion Injury

Mast cells play critical roles in I/R injury across multiple organ systems, contributing to both the initial tissue damage and subsequent inflammatory responses [148]. I/R injury is a complex clinical issue caused by organ ischemia and subsequent blood supply restoration. During the ischemic phase, tissue hypoxia and metabolic stress can activate MCs through multiple mechanisms, including adenosine receptor stimulation and reactive oxygen species generation [149].

The reperfusion phase is characterized by massive MC degranulation and inflammatory mediator release. Histamine release contributes to increased vascular permeability and edema formation, while protease release promotes complement activation and inflammatory cell recruitment. The generation of lipid mediators during reperfusion amplifies the inflammatory response and contributes to tissue damage. MC involvement in I/R injury is unclear, but chemicals released by MCs can trigger vasoactive substance formation, tissue leakage, and leukocyte recruitment. Long-term, MCs may influence tissue remodeling and blood restoration. Targeting MCs has been shown to attenuate I/R injury [149]. The temporal pattern of MC activation during I/R injury involves both immediate degranulation and prolonged inflammatory mediator synthesis. This biphasic response contributes to both acute tissue damage and chronic remodeling processes that can lead to organ dysfunction.

An earlier study comparing cardiac tissue susceptibility and serum IL-6 changes between MC-deficient (W/Wv) and normal littermates revealed that the absence of MCs reduces myocardial damage associated with I/R injury. Serum IL-6 levels were higher in the +/+ sham mice than in the W/Wv mice. The absence of MCs also attenuates the inflammatory response, as measured by serum IL-6 levels, following the local insult [150]. In fact, it was shown that immobilization stress induced MC-derived release of IL-6 in mice [133]. This finding suggests the possibility of developing prophylactic therapy targeting selective inhibition of cardiac MC activation in clinical situations involving myocardial revascularization [150].

#### 3.3.3. Cardiovascular Diseases

Cardiovascular diseases are the leading cause of mortality and disability globally, increasing since 1990 due to changing risk factors, population growth, and aging [151]. This study estimated global, national, and subnational CVD burden, including 18 subdiseases and 12 risk factors, including family history, diabetes, obesity, hypertension, and stress, as well as high homocysteine and IL-6 levels. In spite of the well-known effect of lipid build-up and occlusion of the coronary arteries, coronary inflammation in response to atheromas or other drivers has emerged as probably the most important pathogenetic mechanism in CVD and CAD [152,153,154,155], leading to a new field of cardiology, “Atheroimmunology” [156].

**Figure 2:**
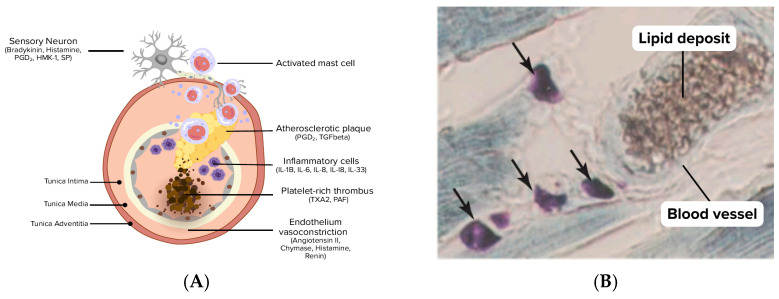
(**A**) Diagrammatic representation of a coronary artery (cross section) showing mast cells involved in processes contributing to cholesterol build-up, inflammatory cell accumulation, coronary constriction, and coronary nerve sensitization contributing to CAD. The molecules in parentheses indicate mast cell-derived mediators associated with distinct pathologic processes. (**B**) Photomicrograph of mast cells stained with toluidine blue (arrows) adjacent to a coronary blood vessel (bv), occluded by lipid deposits stained with Sudan black (magnification = ×1000).

Mast cells are also found in the human heart and atherosclerotic plaques [51,52,54,157,158,159], producing various vasoactive and proinflammatory mediators that affect inflammation, angiogenesis, lymphangiogenesis, tissue remodeling, and fibrosis [144,145]. MCs are involved in non-allergic disorders like atherosclerosis, contributing to neovascularization, plaque progression, instability, erosion, rupture, and thrombosis [160]. The phenotype of cardiac MCs appears to be different from other tissue MCs, as they do not respond to morphine and substance P (SP), a strong trigger of skin MCs [50,53], see Figure 2.

Secretion of vasoactive mediators, cytokines, and proteinases contributes to plaque progression, diet-induced obesity, and diabetes. Understanding these processes could lead to novel therapeutic applications [15]. MCs play a crucial role in cardiac homeostasis and inflammation. Their origin, development, and replenishment in the heart are underexplored [161]. The involvement of MCs in cardiovascular pathology extends beyond acute allergic reactions like Kounis syndrome to include chronic conditions such as atherosclerosis, heart failure, and hypertension [14]. MCs are found in atherosclerotic plaques, where they contribute to plaque instability through protease release and inflammatory mediator production [14]. Cardiac MCs are involved in tissue homeostasis and disease [162]. Autoantibodies to IgE and/or FcεRI have been found in patients with various immune disorders. Functional human IgG anti-IgE and rabbit IgG anti-IgE induced the release of proinflammatory mediators from cardiac MCs, with human monoclonal IgE acting as a competitive antagonist [163].

Mast cells are also linked to an increased prevalence of CVD in patients with mastocytosis, contributing to plaque formation, plaque instability, and fibrosis formation after cardiac infarctions, mastocytosis, and allergy [162].

Atherosclerosis is a progressive disease caused by endothelial dysfunction and inflammation. The vascular endothelium, responsible for detecting changes in hemodynamic forces, releases vasoactive substances. Disruption of this balance, mediated by inflammatory and cardiovascular risk factors, leads to atheroma formation. Inflammatory mediators play a crucial role in atherosclerotic plaque formation. Markers of inflammation and endothelial activation could provide additional information about a patient’s risk of cardiovascular disease and new treatment targets [125].

Commonly, coronary artery disease (CAD) necessitates surgical intervention like coronary bypass grafting and percutaneous coronary interventions. Restenosis, caused by endothelial damage, is a significant burden of mortality and morbidity. MCs play a role in atherosclerosis and vascular diseases, but their rapid response to arterial wire injury can cause restenosis. Targeting MC degranulation with disodium cromoglycate could reduce restenosis and prevent further complications [164].

Mast cells contribute to atherosclerotic plaque inflammatory processes [159], potentially indicating increased MC tryptase levels as a biomarker in patients with stable coronary artery disease (CAD) [129]. A study found that patients with significant CAD had significantly higher serum tryptase levels, a 4.3-fold risk. Tryptase levels may be a result of chronic low-grade inflammatory activity in atherosclerotic plaques, potentially serving as a new biomarker for identifying asymptomatic CAD patients [128].

One study investigated the relationship between peripheral levels of tryptase, an MC-specific protease, and left ventricular size and function, congestive heart failure (CHF), and CAD. Results showed that systemic tryptase levels were lower in patients with increased left ventricular end-diastolic volume (LVEDV), CHF, and depressed left ventricular ejection fraction (LVEF). There was no significant relationship between tryptase levels and LVEF or LVEDV. The presence of CAD was associated with elevated systemic tryptase levels [165].

Chymase-mediated angiotensin II generation in vascular tissues contributes to hypertension and vascular remodeling. The local production of angiotensin II by MC chymase can occur independently of the circulating renin-angiotensin system, providing an additional mechanism for blood pressure regulation [166].

In heart failure, MC activation contributes to cardiac fibrosis and remodeling through the release of fibrogenic mediators. Histamine and other MC mediators can directly affect cardiac contractility and rhythm (Table 3), potentially contributing to arrhythmias and sudden cardiac death [14].

The microvascular effects of MC activation in CAD include endothelial dysfunction, increased permeability, and inflammatory cell recruitment. These effects contribute to the progression of cardiovascular pathology and can influence clinical outcomes [14].

Stress can activate MCs’ asthma, atopic dermatitis, and acute coronary syndromes (ACSs), which are linked to coronary inflammation. Stress activates coronary MCs through corticotropin-releasing hormone (CRH) and other neuropeptides, contributing to coronary inflammation and coronary artery disease. This leads to hypersensitivity, inflammation, and coronary hypersensitivity. The presence of atherosclerosis increases the risk of cardiac MC activation due to the stimulatory effect of lipoproteins and adipocytokines. Conditions like Kounis syndrome, mastocytosis, and myalgic encephalopathy/chronic fatigue syndrome are particularly prone to coronary hypersensitivity [167].

Cardiac MCs [168,169] are activated in atherosclerotic coronary arteries by releasing proinflammatory cytokines [170]. MC activation has been associated with coronary artery injury [164]. At least one case of mastocytosis has been associated with “pulseless-electrical-activity” cardiac effects resistant to cardiac pacemakers [171]. Interestingly, the use of MC stabilizers in mouse models prevented arrhythmogenesis [172]. A 2002 study investigated the effect of acute stress on cardiac MC activation and histamine levels in normal and genetically deficient mice. Results showed a significant reduction in cardiac histamine and an increase in serum histamine in ApoE k/o mice. The high basal cardiac and serum histamine levels in ApoE k/o mice suggest ongoing cardiac MC activation that may contribute to atherosclerosis, potentially helping to understand stress-related cardiovascular pathology [132].

Another study examining stress-induced IL-6 release in atherosclerotic mice found that it is influenced by MCs, corticotropin-releasing hormone (CRH), and urocortin (Ucn). The study found that stress-induced IL-6 release was more pronounced in apolipoprotein E (ApoE) knockout (k/o) atherosclerotic mice, with MCs found adjacent to atherosclerotic vessels. Stress-induced IL-6 release was partially inhibited by cromolyn in C57BL and ApoE mice. The findings suggest that stress-induced IL-6 release may be partly due to peripheral Ucn and/or CRH, possibly overcompensating for CRH deficiency [133].

One more study investigated the role of MCs in the physiological aging of the heart and kidney. It compared the number of MCs in the left and right heart ventricles and kidneys of Wistar rats and evaluated the immunohistochemical expression of basic fibroblast growth factor. The results showed that the number of MCs increases significantly in older rats, with the greatest increase in the left ventricle, followed by the right ventricle and the kidney [17].

Mast cells have been involved in cardiac fibrosis [173]. A recent study aimed to develop a minimally invasive, targeted, and convenient vagus nerve stimulation (VNS) approach to assess the impact of VNS on the prognosis of patients with myocardial atrophy after acute ischemic stroke. Using a rat model of middle cerebral artery occlusion, the researchers found that VNS improved myocardial atrophy, inhibited MC activation, reduced the expression of chymase and angiotensin II (AngII), and inhibited the expression of proinflammatory factors. The study suggests that inhibiting MC activation may be an effective strategy for treating myocardial atrophy after stroke [174] and cardiac remodeling [175], as it reduces the expression of chymase and AngII [176].

#### 3.3.4. Heart–Brain Axis: Role of Stress and CRH Peptides in Mast Cell-Mediated Microvascular Inflammation

A growing body of evidence identifies a functional heart-brain axis (HBA) in which psychological and physical stress directly activates cardiac MCs through neuroendocrine peptides, most prominently corticotropin-releasing hormone (CRH). The HBA constitutes a bidirectional physiological network integrating neural regulation of cardiovascular function with cardiovascular influences on brain health, operating through autonomic nerves, endocrine signaling, and immune mediators [177,178,179,180].

Under acute stress, the paraventricular nucleus (PVN) of the hypothalamus releases CRH, orchestrating the classical hypothalamic–pituitary–adrenal (HPA) axis, while simultaneously exerting potent pro-inflammatory peripheral effects through direct activation of CRH receptors (CRHR-1 and CRHR-2) expressed on cardiac MCs. Seminal animal studies demonstrated that acute immobilization stress induces marked cardiac MC degranulation, an effect blocked by the CRH-receptor antagonist Astressin and by the MC stabilizer cromoglycate (cromolyn), while a neurotensin receptor antagonist also inhibited this response, implicating local CRH and co-released neurotensin (NT) as primary neuroimmune triggers of coronary MC activation [132,181]. Cardiac histamine release triggered by restraint stress was confirmed to be strictly MC-dependent, absent in mast cell-deficient W/W_v_ mice, and significantly amplified in atherosclerosis-prone ApoE knockout mice, demonstrating a synergistic interaction between stress-induced MC activation and pre-existing cardiovascular risk [132,181].

CRH does not act alone in this pathway. It is frequently co-released with NT and urocortin (Ucn), a structurally related peptide that triggers rat skin and coronary MC degranulation and vascular permeability at nanomolar concentrations equipotent to calcitonin gene-related peptide and NT, an effect abolished by cromolyn and absent in MC-deficient mice, with histamine identified as the principal vasoactive mediator released [181,182]. CRHR-2β, predominantly expressed in the left ventricle and cardiomyocytes, provides anatomical specificity for direct stress-peptide cardiotoxic effects, whereas CRHR-1 selectively mediates CRH-induced VEGF secretion from human MCs without classical degranulation, establishing a distinct angiogenic and pro-permeability pathway [167,182]. The expression of CRHR-2 mRNA and protein on human cord-blood-derived MCs is inducible by IL-4 and further upregulated by IL-1 and LPS, indicating that inflammatory priming enhances MC sensitivity to CRH signals and may amplify the HBA neuroimmune loop in the context of pre-existing cardiovascular inflammation [167]. Once activated, coronary MCs release histamine, tryptase, IL-6, TNF-α, and VEGF, triggering endothelial dysfunction, microvascular permeability, platelet activation, and coronary hypersensitivity reactions that can precipitate acute coronary syndromes (ACS). Recent cardiovascular pharmacology evidence further demonstrates that MC-derived tryptase and chymase convert angiotensin I to angiotensin II independently of ACE, directly driving cardiac fibrosis and adverse remodeling, while MC-derived histamine stimulates H2 receptors on sinoatrial cardiomyocytes to induce chronotropic effects and arrhythmias [183,184].

Beyond acute psychological stress, structural brain injury similarly activates this axis. A recent systematic review and meta-analysis confirmed that traumatic brain injury (TBI) independently raises the risk of cardiovascular disease by approximately threefold, mediated through sustained HPA axis dysregulation, autonomic nervous system imbalance, and chronically elevated CRH signaling [185]. Atrial fibrillation (AF) represents a clinically important bidirectional node of the HBA: AF increases relative dementia risk 1.4–2.2-fold independently of clinical stroke, through mechanisms including silent cerebral infarction (present in 25–40% of AF patients), cerebral hypoperfusion, and systemic neuroinflammation; conversely, brain insults drive AF through neurogenic autonomic surges [178]. Stress-responsive hormonal pathways, including the HPA axis, the sympathetic-adrenal medullary system, and the BDNF/TrkB pathway, contribute to bidirectional heart-brain communication, with chronic dysregulation implicated in vascular injury, mood disorders, and accelerated cognitive decline [177]. Sympathetic activation and brain injury both trigger systemic immune responses with downstream effects on the myocardium and cerebral vasculature: inflammatory cytokines, microvesicles, and oxidative stress contribute to endothelial dysfunction, blood–brain barrier disruption, and altered neurovascular coupling, further perpetuating the HBA neuroimmune cascade [186].

Human MCs themselves synthesize and secrete CRH and Ucn, establishing an autocrine–paracrine amplification loop in which MC-derived CRH further activates neighboring MCs and potentiates local vascular inflammation independently of central hypothalamic drive. In mast cell activation disease (MCAD), this amplification is clinically manifest as non-cardiac angiopectoris, coronary vasospasm, Raynaud’s phenomenon, arrhythmias, and—in severe cases—sudden cardiac arrest, all attributable to inappropriate MC mediator release [187,188]. In heart failure with preserved ejection fraction (HFpEF), accumulating evidence implicates MC-mediated microvascular inflammation, including IL-1 signaling, tryptase-driven fibrosis, and coronary microvascular dysfunction, as pivotal pathophysiological contributors, with MC stabilization emerging as a promising immunomodulatory target [177,189]. More broadly, mast cells in the cardiovascular system regulate fibrosis through tryptase and chymase, angiogenesis via VEGF-driven pathways, and atherogenesis through plaque destabilization and lipid accumulation, forming a central effector arm of the HBA [25,190].

**Figure 3:**
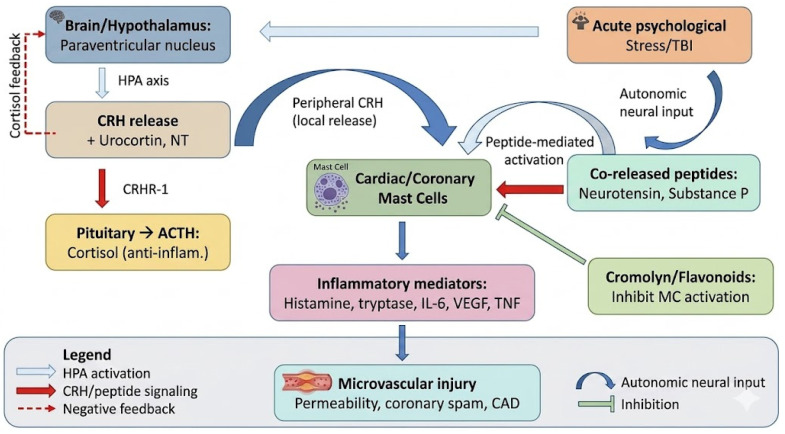
Heart-Brain Axis: CRH/Neuropeptide-mediated mast cell activation in coronary microvascular inflammation. Psychological and physical stress and traumatic brain injury TBI → hypothalamic PVN → CRm/NT/Ucn release → CRHR-1/CRHR-2 on cardiac MCs → histamine, tryptase, IL-6, TNF-α, VEGF → microvascular injury/CAD. Autocrine CRH loop shown as returning arrow from MC back to MC. Cortisol negative HPA feedback shown in dashed line. Flavonoid/cromoglycate inhibition shown in green block. Designed using PowerPoint and Gemini-AI.

Therapeutically, the HBA offers multiple intervention points. Pharmacological blockade of CRHR-1 with the selective non-peptide antagonist antalarmin, or of NT receptors, interrupts stress-triggered cardiac MC degranulation in vivo, as does pretreatment with the MC stabilizer cromoglycate [132,181]. Recent evidence shows that MC-stabilizing drugs should be considered a first-line approach in MCAD-related cardiovascular manifestations and that inhibition of platelet activation blunts MC-dependent microvascular inflammation following cardiac surgery [179,180]. Flavonoids are particularly relevant here: their established inhibitory effects on CRHR-1 downstream signaling, calcium mobilization, and NF-κB activation in MCs position them as natural compounds capable of targeting the neuroimmune bridge between psychological stress and coronary microvascular pathology. Comprehensive risk-factor management, including blood pressure control, lifestyle interventions, and treatment of sleep apnea, can attenuate neuroinflammation and reduce the chronically elevated HPA axis activity that sensitizes cardiac MCs to future stress-triggered degranulation [177,178]. Collectively, these findings establish the HBA, operating through CRH, NT, and Ucn signaling to coronary MC CRHR-1/CRHR-2, as a mechanistically coherent and therapeutically tractable pathway linking psychological and neurological stress to microvascular inflammation and cardiovascular disease (see Figure 3).

#### 3.3.5. COVID-Related Cardiomyopathy

Mast cells play important roles in neuroinflammatory conditions through their ability to cross-talk with the nervous system and influence BBB function [191]. These cells are found in close proximity to the BBB, where they can respond to both systemic and CNS stimuli. MCs are crucial for immunity and inflammation but may also be involved in dysautonomias and neuroinflammatory disorders. They are located near nerve endings and can be affected by the autonomic nervous system (ANS) [192]. MCs can regulate dysfunctional homeostatic functions, especially in COVID-19 and Long COVID syndrome [141,193,194].

Cardiomyopathy has emerged as a significant symptom associated with COVID-19, Long COVID, and following COVID vaccination [195,196,197]. COVID-associated myocardial injury has been associated with local inflammation [198]. MC activation may explain cardiac pathology in such cases [199]. For instance, the derived histamine could cause coronary vasoconstriction, leading to cardiomyocyte anoxia [200]. The release of inflammatory mediators promotes neuronal damage, glial activation, and BBB breakdown. Mast cell stabilization has shown protective effects in experimental models of neurological injury [62,201].

The interaction between MCs and the nervous system involves bidirectional communication through neuropeptides, neurotransmitters, and growth factors. This neuro-immune cross-talk can influence both MC function and neuronal responses [62,201].

The COVID-19 pandemic has significantly impacted CVD, with acute and post-acute complications causing long-term sequelae. The European Society of Cardiology has developed a clinical consensus statement addressing CVD prevention strategies, including acute infection, prior infection, Long COVID, reinfection, and post-vaccination events [202]. Key recommendations include preventing and managing cardiovascular manifestations, implementing targeted rehabilitation, and introducing interventions to mitigate Long COVID severity. Future research should focus on individualizing preventive measures and refining rehabilitation strategies. This Medical News article is an interview with the lead author [203]. Table 4 depicts the MC involvement across various inflammatory disease conditions affecting microvasculature (Objective 3).

## 4. Therapeutic Interventions

The main treatment approach for CVD is to reduce or eliminate as many risk factors as possible. Reduction in cholesterol has been the main target of the use of statins [204]. However, these HMG-CoA reductase inhibitors have been shown to owe their benefits to additional actions [205,206,207], especially their immunomodulating effects [208]. However, statins have been associated with myopathies, limiting their usefulness over prolonged periods [208]. As a result, recent approaches have focused on reducing cardiovascular inflammation not only in CVD [154,209,210] but also in atrial fibrillation [211,212,213]. However, there are no effective anti-inflammatory drugs except for glucocorticoids, which are associated with detrimental effects on the heart [214] or biologics that are linked to increased risk of infection and cancer [215].

Interventions to modulate MC activation are a promising approach for the prevention or treatment of CVD [14,168]. In this context, the use of naturally occurring flavonoids is of particular interest [32].

### 4.1. Flavonoids: Structure, Classification, and Bioavailability

#### 4.1.1. Chemical Structure and Classification

Flavonoids share a common C6-C3-C6 carbon skeleton consisting of two aromatic rings (A and B) connected by a three-carbon bridge that forms a heterocyclic ring (C). This basic structure provides the framework for extensive structural diversity through various substitution patterns, hydroxylation, methylation, and glycosylation reactions [24].

The major subclasses of flavonoids include flavonols (quercetin, kaempferol, myricetin, and pycnogenol), flavones (apigenin and luteolin), flavanones (hesperidin and naringenin), flavan-3-ols (catechin, epicatechin, and eriodictyol), anthocyanins (cyanidin and delphinidin), isoflavones (genistein and daidzein), and methylated flavones such as tetramethoxyluteolin and hexamethoxyflavone (nobiletin). Each subclass exhibits distinct structural features and biological activities [24,216]. As an example, a systematic review and meta-analysis of randomized controlled trials (RCTs) found that pycnogenol supplementation may have a role in preventing cardiometabolic disease. The meta-analysis included 24 RCTs, including 1594 participants, and found that pycnogenol significantly reduced fasting blood glucose, glycated hemoglobin, systolic blood pressure, diastolic blood pressure, body mass index, and LDL cholesterol and increased HDL cholesterol [217].

Structure–activity relationships have revealed that specific structural features influence the biological activities of flavonoids. The presence and position of hydroxyl groups, the degree of hydroxylation, and the substitution pattern on the B ring are particularly important for antioxidant and anti-inflammatory activities. The 3’,4’-dihydroxy substitution on the B ring (catechol group) is associated with enhanced antioxidant activity [24].

Glycosylation significantly affects the biological properties of flavonoids, generally reducing their antioxidant activity but potentially enhancing their stability and bioavailability. The aglycone forms are typically more potent in biological assays but may have limited absorption and distribution characteristics [218].

#### 4.1.2. Dietary Sources and Intake

Flavonoids are widely distributed in plant foods, with particularly high concentrations found in fruits, vegetables, tea, coffee, wine, and chocolate [26]. Onions, apples, berries, citrus fruits, and leafy green vegetables are among the richest dietary sources of flavonoids. Tea consumption provides significant amounts of flavan-3-ols, while red wine is a major source of anthocyanins and other flavonoids [219]. Olive tree components contain a number of polyphenolic compounds, including oleuropein, hydroxytyrosol, and luteolin [220]. Flavonoids have been particularly attractive for cardiovascular disorders [26,221].

The flavonoid content of foods can vary significantly depending on genetic factors, growing conditions, processing methods, and storage conditions. Environmental stressors such as UV radiation, temperature extremes, and pathogen attack can increase flavonoid production in plants as protective mechanisms [222]. Estimated daily flavonoid intake varies considerably among populations, ranging from less than 100 mg to over 1000 mg per day. Mediterranean and Asian populations typically have higher flavonoid intakes due to greater consumption of fruits, vegetables, and tea. Processing and cooking can affect flavonoid content, with some methods causing losses while others may increase bioavailability [223].

Traditional medicinal plants represent reasonable sources of specific flavonoids and have been used for centuries to treat inflammatory conditions. Modern research has validated many of these traditional uses and identified the active flavonoid compounds responsible for therapeutic effects [222].

#### 4.1.3. Absorption, Metabolism, and Bioavailability

The bioavailability of flavonoids is generally low, with absorption rates typically ranging from 1 to 10% for most compounds. Glycosylated flavonoids require deglycosylation by intestinal or bacterial enzymes before absorption, while aglycones can be directly absorbed through passive diffusion or active transport mechanisms [224].

The small intestine is the primary site of flavonoid absorption, though some compounds can be absorbed in the colon following bacterial metabolism. Specific transporters, including glucose transporters (GLUT) and organic anion transporters, facilitate the uptake of certain flavonoids [225].

Extensive first-pass metabolism occurs in the intestine and liver, involving phase I (hydroxylation, demethylation) and phase II (glucuronidation, sulfation, methylation) enzymatic reactions. These metabolic transformations generally reduce the biological activity of flavonoids but enhance their water solubility and excretion [225].

Factors affecting bioavailability include food matrix effects, individual genetic variations in metabolizing enzymes, gut microbiota composition, and co-ingestion with other nutrients. Certain food components can enhance flavonoid absorption, while others may inhibit it. Understanding these factors is crucial for optimizing therapeutic applications of flavonoids [224,226]. Table 5 depicts the structural characteristics and pharmacokinetic properties of therapeutically relevant flavonoids (Objective 4).

### 4.2. Antioxidant, Anti-Allergic, and Anti-Inflammatory Effects of Flavonoids

#### 4.2.1. Antioxidant Properties

Oxidative stress, a physiological level of reactive oxygen species (ROS) and reactive nitrogen species (RNS), can be harmful or beneficial to biological systems. The oxygen/nitrogen free radicals and non-radical reactive species (collectively known as ROS/RNS) are termed oxidative eustress or “good stress”. Oxidative stress, an imbalance between ROS/RNS production and elimination, is a common cause of cancer, cardiovascular diseases, diabetes, neurological disorders, psychiatric disorders, renal disease, lung disease, and aging. Organisms have evolved a complex antioxidant defense system to counteract the harmful effects of ROS. The first-line defense mechanism involves enzymes like superoxide dismutase (SOD), catalase (CAT), and glutathione peroxidase (GPx), which play a crucial role in the dismutation of superoxide radicals and hydrogen peroxide. The second-line defense pathway involves exogenous diet-derived small-molecule antioxidants. The third-line defense ensures the repair or removal of oxidized proteins and other biomolecules.

Oxidative stress contributes to the pathology of many diseases through the chemistry of ROS and RNS, their role in oxidative damage of DNA, proteins, and membrane lipids, and their potential use in pharmaceutical interventions—redox metal-based enzyme mimetic compounds and sirtuins. The antioxidant properties of flavonoids contribute significantly to their anti-inflammatory effects. ROS plays important roles in MC activation and inflammatory mediator release, making antioxidant intervention therapeutically relevant [232,233]. They are also promising therapeutic targets for age-related diseases and anti-aging strategies [232]. Flavonoids scavenge various ROS, including superoxide anions, hydroxyl radicals, and peroxynitrite, through direct radical quenching mechanisms. The catechol groups present in many flavonoids are particularly effective for radical scavenging through electron donation.

Flavonoids can upregulate endogenous antioxidant systems, including catalase, superoxide dismutase, and glutathione peroxidase. This enhancement of cellular antioxidant defenses provides sustained protection against oxidative stress. The activation of the Nrf2 (nuclear factor erythroid 2-related factor 2) pathway by flavonoids contributes to antioxidant enzyme induction [233].

#### 4.2.2. Inhibition of Mast Cell Degranulation and Mediator Secretion

Certain natural flavonoids have potent antioxidant and anti-inflammatory properties [26,234] and can also modulate PAF [235]. Flavonoids exert potent inhibitory effects on MC degranulation through multiple mechanisms involving calcium signaling, membrane stability, and protein kinase modulation. Quercetin, the most extensively studied flavonoid in this context, inhibits histamine release from MCs at concentrations achievable through dietary intake. Quercetin is a flavonol found in vegetables, fruits, herbs, tea, and wine, known for its antioxidant and anti-allergic properties [236]. Quercetin’s plant extract is used in anti-allergic drugs, supplements, and enriched products, effectively inhibiting IL-8 and suppressing IL-6 and calcium levels [237]. Both quercetin [238] and the flavone luteolin [239] were more effective than the “MC stabilizer” cromolyn in inhibiting activation of human MCs. Luteolin inhibited activation of both human MCs [122,240] and brain microglia [241,242]. Luteolin not only inhibits MC activation but also neuroinflammation [243,244]. Moreover, luteolin is neuroprotective [243,245] and could reduce cognitive dysfunction [246,247], especially brain fog [248]. Hence, luteolin has been termed “the wonder flavonoid” [249] and has immunopharmacological potential for many diseases [250]. The luteolin structural analog tetramethoxyluteolin (methoxyluteolin) is even more potent than luteolin [122,238,242] and has been incorporated into a skin lotion [251].

Beyond their effects on degranulation, flavonoids potently inhibit the synthesis of inflammatory mediators by activated MCs. The suppression of arachidonic acid (AA) metabolism represents a major target, with flavonoids inhibiting both cyclooxygenase and lipoxygenase pathways. AA, an n-6 essential fatty acid, is crucial for mammalian cells and plays a role in the progression of diseases like hepatic fibrosis, neurodegeneration, obesity, diabetes, and cancers. It is the precursor of eicosanoids, which trigger oxidative stress and stimulate the immune response. Interventions in AA metabolic pathways are effective in managing inflammation-related diseases. Current drug discovery focuses on AA metabolism and its potential clinical applications [131]. Flavonoids can also inhibit the synthesis and action of PAF [130,235,252,253].

Quercetin and other flavonoids inhibit phospholipase A2 (PLA2), the rate-limiting enzyme for AA release from membrane phospholipids. This inhibition reduces the substrate availability for downstream inflammatory mediator synthesis. Additionally, direct inhibition of cyclooxygenase-2 (COX-2) and 5-lipoxygenase (5-LOX) by flavonoids further suppresses prostaglandin and leukotriene production. The following study explored the potential of flavonoids as an alternative to traditional analgesics and anti-inflammatory agents, particularly those with dual-inhibitory action on COX-2 and 5-LOX. The researchers predicted drug-likeness properties of synthesized flavonoids using Lipinski’s Rule of Five and evaluated their inhibitory activities using enzyme assays. The results showed that the compounds known as NPC6 and NPC7 showed better selectivity towards COX-2 and 5-LOX, with similar binding modes to Zileuton in the active site of 5-LOX [254].

#### 4.2.3. Specific Flavonoid Compounds and Their Mechanisms

Pycnogenol is often used as an antioxidant and vasodilator [216] with benefits in CVD [217].

Quercetin, the most abundant dietary flavonoid, found in fruits and vegetables, has numerous health benefits, including antioxidant, antimicrobial, anti-inflammatory, antiviral, and anticancer properties. It also exhibits potent MC-stabilizing properties through multiple mechanisms. Its ability to inhibit histamine release, suppress cytokine production, and scavenge reactive oxygen species makes it particularly effective for treating inflammatory conditions. Clinical studies have demonstrated the anti-inflammatory effects of quercetin supplementation in various conditions. Quercetin also has cardiovascular benefits, such as lowering blood pressure and improving endothelial function. Future applications in nutraceuticals, pharmaceuticals, and functional foods are promising, but further research is needed to understand their mechanisms and safety [255].

Luteolin, a flavonoid with antioxidant, anti-tumor, and anti-inflammatory properties, has been shown to protect against CVD in vitro and in vivo. Understanding luteolin’s cardioprotective mechanisms could lead to new drug design and development for CVD [256,257,258]. Recent studies suggest that luteolin suppresses systemic and neuroinflammatory responses in COVID-19 and Long COVID [249,259,260], improving cognitive decline and enhancing neuroprotection in neurodegenerative diseases, traumatic brain injury (TBI), and stroke [192,244].

Fisetin, found in strawberries and other fruits, also shows potent MC-stabilizing activity. It shields cells from oxidative stress, potentially aiding in managing diseases like arthritis and cardiovascular disorders. Its ability to cross the BBB makes it particularly interesting for treating neuroinflammatory conditions. Fisetin also improves cognitive functions and mitigates oxidative damage in neurodegenerative diseases. Animal studies validate its therapeutic potential, showing enhanced memory, reduced neuroinflammation, and increased brain-derived neurotrophic factor levels.

Hesperidin and diosmin, abundant in citrus fruits, exhibit specific effects on microvascular protection [261]. These flavonoids strengthen capillary walls, reduce vascular permeability, and inhibit inflammatory cell infiltration. Diosmin, a flavone glycoside, has been shown to have antioxidative, antihyperglycemic, anti-inflammatory, antimutagenic, and anti-ulcer properties. Diosmin and hesperidin significantly improved motor performance, nerve conduction, and compound muscle action potential amplitude in diabetic rats. These effects were linked to modulation of the FGF21 and galectin-3 pathway, suggesting potential as adjunctive therapies for diabetic neuropathy [261]. Additional clinical studies have demonstrated the efficacy of diosmin and hesperidin in treating chronic venous insufficiency and other vascular disorders [262].

Kaempferol and myricetin [263] show potent anti-inflammatory effects through NF-κB pathway inhibition and antioxidant activities. The following study investigated the antioxidant and anti-inflammatory effects of Kaempferol-3-O-β-D-glucuronate (K3G), a natural flavonoid glycoside, against lipopolysaccharide-stimulated BV2 microglial cells. The results show that K3G inhibits the release of nitric oxide, IL-6, and TNF-α; reduces the expression of pro-inflammatory mediators and cytokines; and downregulates phosphorylated mitogen-activated protein kinases (MAPKs) and upregulates the Nrf2/HO-1 signaling cascade. This results in the inhibition of nitric oxide synthase and the upregulation of antioxidants [264].

Comparative studies indicate that the degree of hydroxylation on the B ring influences both potency and selectivity of anti-inflammatory effects. Myricetin, a natural flavonoid found in vegetables, fruits, nuts, and tea, has antioxidant and anti-inflammatory properties. It helps prevent and treat various diseases by reducing inflammation, maintaining tissue structure, and modulating cell signaling. Myricetin also exhibits anti-microbial properties and synergistic potential with other drugs [265].

We recently showed that the newer flavonoids nobiletin and eriodictyol suppressed the release of IL-1β, CXCL8, IL-6, and MMP-9 from LPS, SARS-CoV-2 spike protein, and ochratoxin A-stimulated human microglia [266]. Table 6 depicts evidence for individual flavonoids in treating MC-mediated inflammatory conditions (Objective 7).

Another polyphenol, the epigallocatechin gallate (EGCG), is the predominant catechin in green tea. It provides protection against UVB-induced carcinogenesis through its anti-inflammatory and antioxidant properties [267,268]. EGCG sits in the flavan-3-ol subclass, structurally adjacent to flavonols but mechanistically distinct, and the gallate ester makes it one of the most structurally complex and bioactive monomeric flavonoids known.

**Table 6:** Specific flavonoids and their therapeutic applications.

Theme/Topic	Key Findings	Studies
Luteolin	Most potent natural MC stabilizer; superior to cromolyn in blocking mediator release; clinical benefits in autism spectrum disorders; neuroprotective in Alzheimer’s disease and brain fog; cardioprotective effects; effective in COVID-19 inflammation	[239,244,248,249,256,257,259,266,269]
Quercetin	Potent anti-allergic effects exceeding cromolyn; inhibits contact dermatitis and photosensitivity; cardiovascular protection; anti-atherosclerotic properties; benefits in metabolic disorders; mortality reduction in cohort studies	[236,237,238,255,270,271,272]
Methoxyluteolin (Tetramethoxyluteolin)	Enhanced bioavailability compared to luteolin; potent mTOR inhibitor in mast cells; effective in autism treatment; benefits in neurodegenerative diseases; topical formulations well-tolerated; inhibits neurotensin and substance P effects	[30,122,240,242,251]
Apigenin	PPARγ activation reducing obesity-related inflammation; macrophage polarization modulation; anti-atherosclerotic effects; mast cell degranulation inhibition	[209,273]
Myricetin	Multi-target pharmacological effects; anti-inflammatory and antioxidant properties; cardiovascular benefits; neuroprotective potential	[263,265]
Kaempferol	Nrf2/HO-1 pathway activation; MAPK/NF-κB inhibition in neuroinflammation; antioxidant effects in microglial activation	[264]
Hesperidin and Diosmin	FGF21 and galectin-3 pathway modulation in diabetic neuropathy; vascular protection; anti-inflammatory in multiple conditions	[261,262]
Pycnogenol (Flavonoid Complex)	Cardiometabolic benefits in meta-analyses; control of inflammation and oxidative stress in chronic diseases; multi-component synergistic effects	[216,217]

### 4.3. Molecular Targets and Signaling Pathways

#### 4.3.1. Direct Protein Interactions

Flavonoids interact directly with numerous proteins involved in inflammatory signaling, providing the molecular basis for their therapeutic effects. Phospholipase A2 (PLA2) represents a major target, with flavonoids binding to the active site and inhibiting enzymatic activity. PLA2 is a key enzyme in inflammation and CVD. A study revealed that resveratrol inhibits PLA2 by reducing sheet content and increasing a 5-helix structure, preventing water molecules from entering the enzyme’s cavity. The binding affinity of resveratrol is thermodynamically sufficient, with van der Waals interactions, particularly hydrophobic ones, playing a significant role in PLA2-resveratrol binding and stability. This information could help improve pharmacological applications [274].

Cyclooxygenase enzymes are directly inhibited by flavonoids through competitive and non-competitive mechanisms. The selectivity for COX-2 over COX-1 exhibited by certain flavonoids provides therapeutic advantages by reducing gastrointestinal side effects. Molecular docking studies have identified key binding interactions that determine selectivity [254].

Ion channel modulation by flavonoids affects cellular excitability and calcium homeostasis. Calcium channels, including L-type voltage-gated channels and store-operated channels, are inhibited by various flavonoids. This channel blockade contributes to the anti-inflammatory effects by preventing calcium-dependent activation processes. Flavonoids specifically target cardiovascular ion channels, which regulate vascular tone and cardiac activity [275].

Protein tyrosine kinases involved in inflammatory signaling are inhibited by flavonoids through ATP-competitive and non-competitive mechanisms. Syk kinase, crucial for MC activation, is potently inhibited by quercetin and other flavonoids. These interactions provide molecular explanations for the observed anti-inflammatory effects. The following study investigated the effects of flavonoids on the NOD-like receptor (NLR) family, pyrin domain-containing 3 (NLRP3) inflammasome pathway, a component of innate immunity. It found that only apigenin, kaempferol, and quercetin significantly inhibited IL-1β production and ASC oligomerization. Apigenin also inhibited the AIM2 inflammasome-related pathway. The action of apigenin on NLRP3 inflammasome activation is mediated partly via inhibition of phosphorylation of the spleen tyrosine kinase/protein tyrosine kinase 2 (Syk/Pyk2) pathway. The study also found that apigenin reduced neutrophils and monocytes in MSU-induced peritonitis in mice [276]. The inhibition of MAP kinase pathways (ERK, p38, and JNK) by flavonoids contributes to reduced cytokine production. These pathways are essential for inflammatory gene transcription and represent important therapeutic targets. Specific flavonoids show selectivity for different MAP kinase pathways, providing opportunities for targeted therapeutic interventions [277].

The inhibition of calcium influx represents a primary mechanism by which flavonoids prevent activation of MCs and other immune cells. Several flavonoids, including quercetin, fisetin, and kaempferol, block calcium channels and prevent the calcium mobilization required for granule–plasma membrane fusion. This effect occurs through direct interaction with calcium channels and modulation of calcium-binding proteins. The following study investigates the effect of natural flavonoids on plasma membrane Ca^2+^-ATPase (PMCA) in isolated protein systems and living cells. Results show quercetin and gossypin are the most potent flavonoids, inhibiting PMCA with different mechanisms [278]. Interestingly, luteolin could inhibit allergic reactions due to activation of both FcεRI- and MRGPRX2 via regulation of calcium signaling [279].

Membrane stabilization properties of flavonoids contribute to their mast cell-stabilizing effects. The lipophilic nature of many flavonoids allows them to intercalate into membrane lipid bilayers, altering membrane fluidity and stability. This stabilization can prevent the membrane fusion events required for degranulation. Flavonoids interact with liposomal membrane structure dependently, decreasing fluidity. The structure-membrane interactivity relationship is characterized by 3-hydroxylation of the C ring, non-modification of the B ring, and 5,7-dihydroxylation of the A ring. Galangin and quercetin inhibit tumor cell proliferation and rigidify cell membranes, but not membrane-inactive flavonoids [231].

Protein kinase C (PKC) inhibition by flavonoids provides another mechanism for preventing MC activation. PKC plays essential roles in both degranulation and cytokine synthesis, making it an important target for anti-inflammatory intervention. Several flavonoids directly inhibit PKC activity, leading to reduced MC responses [280]. Fisetin inhibits MC degranulation through calcium channel blockade and PKC inhibition [281].

#### 4.3.2. Transcriptional Regulation

Cytokine gene expression is significantly reduced by flavonoid treatment through multiple transcriptional and post-transcriptional mechanisms. The nuclear factor kappaB (NF-κB) pathway inhibition represents a primary mechanism, with flavonoids preventing the nuclear translocation and DNA binding of this critical inflammatory transcription factor [277]. This results in reduced transcription of inflammatory genes, including cytokines, chemokines, and adhesion molecules. Recent studies have shown that flavonoids inhibit the NF-κB pathway, affecting common diseases like cancer, cardiovascular, and neurodegenerative ones [282]. For instance, luteolin and methoxyluteolin inhibited NF-κB activation in human MCs [240] and keratinocytes [283].

Activator protein-1 (AP-1) is an inducible transcription factor involved in cell proliferation, migration, and survival. Dysfunctional AP-1 activity is linked to various diseases, including cancer and inflammatory disorders. AP-1 inhibitors can be used in cancer therapy to halt tumor progression. Consuming phytochemicals in the diet has anticancer properties and is related to decreased cancer incidence. Natural product targets AP-1 are effective cancer prevention and treatment options. The AP-1 transcription factor is also targeted by flavonoids, contributing to reduced inflammatory gene expression. The inhibition of AP-1 DNA binding activity by flavonoids occurs through modulation of upstream MAP kinase pathways. This provides an additional mechanism for controlling inflammatory responses [134].

Nuclear factor of activated T cells (NFAT) represents another transcriptional target of flavonoids. NFAT plays important roles in cytokine gene expression and immune cell activation. Flavonoid-mediated inhibition of NFAT occurs through calcineurin pathway modulation [136]. The NFAT family of transcription factors, including NFAT1, NFAT2, and NFAT4, is crucial for T cell activation. They are controlled by calcium influx and costimulatory signaling, increasing IL-2 and IL-2 receptor expression. NFAT proteins interact with other transcription factors and regulate Th cell signature gene expressions. This review discussed recent advances in NFAT-targeting drugs and molecular mechanisms [135].

Peroxisome proliferator-activated receptors (PPARs) are activated by certain flavonoids, leading to anti-inflammatory gene expression programs. Activation by flavonoids promotes resolution of inflammation and tissue repair [273]. This represents a beneficial mechanism distinct from simple inflammatory pathway inhibition. The study reported that apigenin modulated PPARγ, a key regulator of macrophage polarization. It blocks p65 translocation, decreasing NF-κB activation and favoring M2 macrophage polarization. This results in reduced inflammation, improved glucose resistance, and weight loss, making apigenin a potential lead compound for metabolic disorder treatment [273].

#### 4.3.3. Post-Translational Modifications

Protein kinases are crucial for cellular signaling and controlling cell functions. Protein kinase inhibitors (PKIs) are being developed for drug development, with a focus on isoform-selective compounds. Clinical case stories highlight challenges and opportunities in this rapidly evolving field [284]. Protein phosphorylation patterns are significantly altered by flavonoid treatment, affecting signal transduction pathways. Protein kinase inhibition by flavonoids occurs through direct enzyme interaction and competition with ATP binding. This affects the phosphorylation status of key signaling proteins [28]. Flavonols inhibited proinflammatory mediator release, intracellular calcium ion levels, and protein kinase C theta phosphorylation in human mast cells [280]. We had reported that cromolyn [285] and quercetin [286] could influence MC activation by phosphorylating a 78Kd protein, later named mosesin [287]. In fact, activated MCs were associated with the inhibition of a number of kinases [127].

Ubiquitination processes involved in protein degradation and signal regulation are modulated by flavonoids. The ubiquitin-proteasome pathway (UPP) is a crucial system for cellular metabolism, affecting protein degradation and cancer progression. The UPP system plays important roles in inflammatory signaling, particularly in NF-κB pathway regulation. Flavonoids can affect both ubiquitin ligase activity and proteasome function [288,289].

MicroRNA (miRNA) regulation represents an emerging mechanism of flavonoid action on the control of the cell cycle, immune system, mitochondrial dysregulation, inflammation, and angiogenesis. Flavonoids can modulate the expression of specific miRNAs that control inflammatory gene expression. This epigenetic mechanism provides sustained anti-inflammatory effects. Flavonoids can modulate non-coding miRNA function, affecting cancer initiation, growth, proliferation, differentiation, invasion, metastasis, and epithelial-to-mesenchymal transition [290].

Autophagy is a crucial biological process that maintains homeostasis and metabolic balance in cells and inhibits malignant transformation. Autophagy induction by flavonoids contributes to cellular homeostasis and anti-inflammatory effects [291].

#### 4.3.4. mTOR Pathway Modulation

The mechanistic target of the regulatory complex mammalian target of rapamycin (mTOR) is increasingly recognized as an important target for flavonoid action. mTOR regulates cell growth, metabolism, and immune responses, making it relevant for inflammatory conditions. Flavonoids can inhibit mTOR activity through direct and indirect mechanisms as demonstrated in a recent review article on atherosclerosis (AS), a chronic inflammatory disease causing cardiovascular diseases [292]. The mTOR signaling pathway, which regulates autophagy, cell senescence, immune response, and lipid metabolism, is highly correlated with AS risk. Botanical drugs have been found to inhibit the mTOR signaling pathway and delay AS development, providing a new perspective on the mechanisms and precision treatments of AS [292].

The mechanism of luteolin’s inhibition of histamine [122] and IL-31 [293] release may involve inhibiting activation of NfκB or mTOR [122].

Regulatory T cell (Treg) induction by flavonoids occurs partially through mTOR pathway modulation. Tregs play crucial roles in immune tolerance and inflammation resolution. Flavonoid-induced Treg expansion seems to provide a mechanism for sustained anti-inflammatory effects. T lymphocytes, crucial for immune protection and autoimmune diseases, play a role in mTOR pathway activity. Flavonoids can suppress mTOR activity, triggering T regulatory subsets [294].

Metabolic reprogramming induced by flavonoids affects inflammatory cell function and responses. The shift from glycolysis to oxidative metabolism in immune cells reduces inflammatory activation. This metabolic modulation represents a fundamental mechanism of anti-inflammatory action [295].

The immunomodulatory effects of flavonoids extend beyond direct anti-inflammatory actions to include the promotion of immune tolerance. The ability to modulate both innate and adaptive immune responses provides comprehensive anti-inflammatory effects [296]. Table 7 depicts the molecular mechanisms by which flavonoids inhibit MC activation and mediator release (Objectives 5 and 6).

### 4.4. Experimental Evidence and Clinical Studies

#### 4.4.1. In Vitro Studies

Extensive in vitro studies have characterized the effects of flavonoids on MC function using various cell models. Primary MCs isolated from different tissues, immortalized MC lines (RBL-2H3, HMC-1, and LAD2), and human primary MCs have been employed to study flavonoid effects [298].

Dose–response studies have established the concentration ranges required for anti-inflammatory effects. Most flavonoids show significant MC stabilization at concentrations between 10 and 100 μM, with some compounds being effective at lower concentrations. These concentrations are generally achievable through dietary intake or supplementation [299].

Mechanistic investigations using pharmacological inhibitors, gene silencing, and molecular probes have elucidated some of the pathways involved in flavonoid action. The use of calcium imaging, protein kinase assays, and gene expression analysis has provided detailed insights into mechanisms of action. Hyperoside (quercetin 3-O-β-D-galactopyranoside) has shown potential in cancer therapy by targeting multiple mechanisms. Hyperoside induces apoptosis, inhibits proliferation, blocks angiogenesis, and reduces cancer cell metastatic potential. Hyperoside’s potential in managing non-cancerous conditions like diabetes, Alzheimer’s, and pulmonary fibrosis is also explored [300].

The following review explored the inhibitory effects of flavonoids on acetylcholinesterase (AChE), a key target in cognitive impairment treatment. Flavonoids like quercetin, apigenin, kaempferol, and naringenin effectively interact with AChE sites, increasing acetylcholine levels and stabilizing cholinergic signaling, and hold significant therapeutic potential for enhancing cognitive function and treating neurodegenerative diseases [301].

#### 4.4.2. Animal Models

Animal studies have provided crucial evidence for the therapeutic potential of flavonoids in treating inflammatory conditions. I/R models in various organs have demonstrated the protective effects of flavonoid treatment. Reduced tissue damage, inflammatory cell infiltration, and improved organ function have been consistently observed. Aspalathin (ASP), a flavonoid found in rooibos, has been investigated for its potential therapeutic benefits. In vivo studies using two mouse models showed that ASP dose-dependently suppressed immunoglobulin E-mediated passive cutaneous anaphylaxis (PCA) responses and mitigated ovalbumen-induced ASA responses. ASP also reduced IgE-stimulated mast cell degranulation and intracellular calcium influx by inhibiting the FcεRI signaling pathway [302].

Flavonoids can prevent and treat allergic inflammation models, including ovalbumen-induced asthma and PCA. These studies have demonstrated reduced MC activation, decreased inflammatory mediator levels, and improved clinical symptoms [303].

Cardiovascular disease models have revealed protective effects of flavonoids against atherosclerosis, hypertension, and cardiac dysfunction. The mechanisms involve MC stabilization, endothelial protection, and anti-inflammatory effects [304].

Neuroinflammation models have demonstrated the ability of flavonoids to cross the BBB and provide neuroprotective effects. Reduced microglial activation, MC stabilization, and improved neurological outcomes have been reported in models of autism spectrum disorder (ASD) [242]. Flavonoids have been found to alleviate neuroinflammation by inhibiting pro-inflammatory mediators, increasing anti-inflammatory secretion, and modulating microglia and astrocyte polarization mainly via suppressing the activation of the NLRP3 inflammasome, as well as NF-κB, MAPK, and JAK/STAT pathways, promoting Nrf2, AMPK, BDNF/CREB, Wnt/β-Catenin, PI3k/Akt signals, and SIRT1-mediated HMGB1 deacetylation. This review explored their therapeutic benefits and mechanisms, potentially promoting their development into food supplements or lead compounds for neuroinflammation-related brain disorders [305].

#### 4.4.3. Human Clinical Evidence

Cardiovascular outcome studies have demonstrated that higher flavonoid intake is associated with reduced risk of heart disease, stroke, and cardiovascular mortality. These epidemiological findings are supported by intervention studies showing improved endothelial function and reduced inflammatory markers. A meta-analysis of 15 prospective cohort studies found that a high intake of flavonoids is associated with a reduced risk of coronary heart disease and CVD mortality in both men and women. A high intake of flavonoids was also associated with lower total mortality. These findings support the recommendation of high fruit and vegetable intake as part of a healthy diet [271].

Allergic disease studies have shown that flavonoid supplementation can reduce allergic symptoms and improve quality of life. The effects include reduced histamine levels, decreased eosinophil counts, and improved lung function in asthmatic patients. The following systematic review aimed to evaluate the clinical efficacy of flavonoid supplements in treating allergic diseases. A total of 15 randomized controlled trials included 990 participants aged 6 to 69. Twelve studies (80%) showed some benefits of flavonoids in allergic patients, while three (20%) reported no significant impact. No severe adverse events were reported. The review suggested flavonoids may be a viable strategy for mitigating allergic symptoms. Future studies with high methodological quality are needed [306].

Safety profiles of flavonoids have been extensively studied, with most compounds showing excellent safety records [236,307]. Adverse effects are rare and typically mild, making flavonoids attractive candidates for long-term therapeutic use [308]. One such formulation (NeuroProtek^®^) improved children with autism spectrum disorder (ASD) [269], while use of FibroProtek^®^ and BrainGain^®^ was included in the successful treatment of a patient with long COVID [20].

### 4.5. Therapeutic Potential and Drug Development

#### 4.5.1. Structure–Activity Optimization

Lead compound identification efforts have focused on natural flavonoids with potent anti-inflammatory activities and CVD-protective effects [221]. Quercetin, fisetin, and hesperidin have emerged as promising candidates for further development. Structure–activity relationship studies have identified key structural features required for optimal activity. Chemical modifications aimed at improving potency and selectivity have been pursued. Synthetic derivatives with enhanced anti-inflammatory activity and reduced side effects have been developed. These modifications include structural changes to improve stability, bioavailability, and target selectivity. However, clinical applications are limited due to drug delivery and bioavailability issues. Improvements include mechanism-based precision medicine approaches [227]. Selectivity improvements have focused on developing compounds with specific activity against inflammatory pathways while preserving beneficial physiological functions. The development of MC-selective inhibitors represents a promising approach for treating inflammatory conditions.

One review explored the therapeutic potential of prunin, a flavanone glycoside, the aglycone form of which is naringenin, found in immature citrus fruits. The review also explored innovative delivery methods, particularly nanoformulations, to address bioavailability, solubility, and stability limitations. The aim was to stimulate further exploration of using prunin as an anticancer agent, thereby advancing the development of targeted, selective, safe, and effective therapeutic methods [309].

Combination approaches utilizing multiple flavonoids or flavonoids with conventional drugs have shown synergistic effects. These combinations can provide enhanced efficacy while reducing individual compound doses and potential side effects.

#### 4.5.2. Formulation and Delivery Strategies

Bioavailability enhancement techniques have been developed to overcome the poor absorption and extensive metabolism of flavonoids. Nanoparticle formulations, liposomal delivery systems, and cyclodextrin complexation have shown promise for improving flavonoid bioavailability.

Targeted delivery systems have been designed to concentrate flavonoids at sites of inflammation. Nanocarriers functionalized with targeting ligands can selectively deliver flavonoids to activated MCs or inflamed tissues.

Nanotechnology applications include the development of polymeric nanoparticles, solid lipid nanoparticles, and nanoemulsions for flavonoid delivery. These systems can protect flavonoids from degradation, control release rates, and improve tissue penetration. This review discussed the classification of nanoparticles, the advantages of nanoparticle–flavonoid formulations, biomedical applications, safety, and toxicity considerations [230].

Sustained release formulations have been developed to maintain therapeutic flavonoid levels over extended periods. These systems can reduce dosing frequency and improve patient compliance [310]. Integration of South African-origin flavonoids with nanotechnology, presenting a new strategy for improving drug delivery and therapeutic outcomes [297]. Encapsulating flavonoids in nanoparticles could increase solubility and reduce toxic effects [311,312,313]. To improve bioavailability, a quercetin-based nanoformulation has been considered, enhancing uptake by the epithelial system and delivery to the target site [272].

Luteolin, like most flavonoids, is absorbed less than 10% from the gut; however, when luteolin is formulated in a liposomal form using olive pomace oil [314] alone (e.g., PureLut^®^) or together with quercetin (e.g., FibroProtek^®^), it increases absorption considerably and also provides the well-known benefits of olive oil [220]. One such formulation (NeuroProtek^®^) improved children with autism spectrum disorder (ASD) [269], while the use of FibroProtek^®^ and BrainGain^®^ was included in the successful treatment of a patient with long COVID [20].

#### 4.5.3. Combination Therapies

Synergistic effects with conventional anti-inflammatory drugs have been demonstrated for several flavonoids. The combination of quercetin with corticosteroids has shown enhanced anti-inflammatory effects with reduced steroid doses. Similar synergistic effects have been observed with antihistamines and other conventional therapies [270,315]. Multi-target therapeutic approaches utilizing flavonoids address the complex nature of inflammatory diseases. The ability of flavonoids to simultaneously target multiple inflammatory pathways provides advantages over single-target therapies.

**Figure 4:**
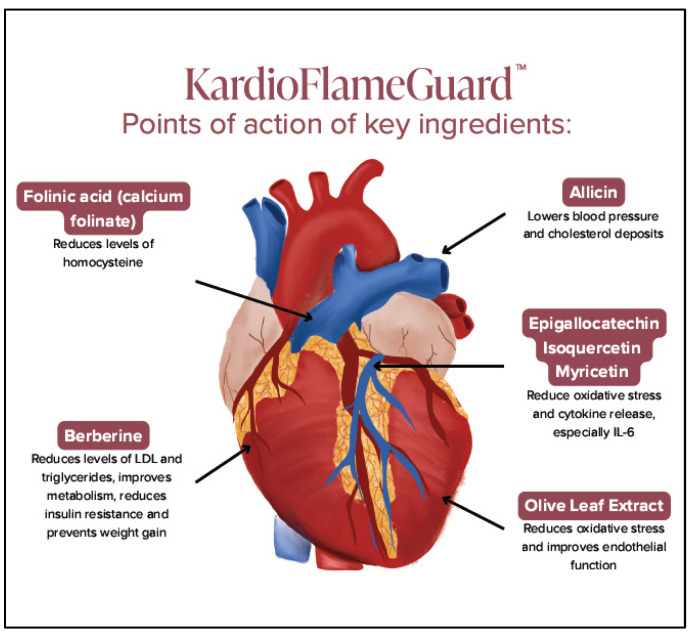
Diagrammatic representation of points of action of natural molecules in the dietary supplement KardioFlameGuard.

A novel dietary supplement (KardioFlameGurad^TM^) combines several natural molecules, including certain flavonoids (Figure 4), aimed to reduce a number of CVD risk factors, leading to synergistic benefits. As an example, berberine can improve obesity and hyperlipidemia by decreasing triglycerides (TG), total cholesterol (TC), and low-density lipoprotein (LDL), while elevating high-density lipoprotein (HDL). It also enhances insulin sensitivity to combat type 2 diabetes and offers protection against diabetic encephalopathy [316]. The clinical efficacy and safety of berberine were examined in non-alcoholic fatty liver disease (NAFLD), which is becoming increasingly prevalent globally. The following meta-analysis evaluated berberine’s effectiveness and safety in treating NAFLD by analyzing 10 RCTs with 811 patients. Significant reductions were observed in liver function markers (ALT, AST, GGT), lipid indices (TG, TC, LDL-C), insulin resistance (HOMA-IR), and body mass index (BMI), indicating berberine’s efficacy. Furthermore, it presented a favorable safety profile with only mild gastrointestinal adverse events noted. The findings suggest that berberine may serve as a promising adjunct therapy for NAFLD [317].

Personalized medicine focuses on tailoring therapies, disease prevention, and health maintenance to individual needs. Pharmacogenomics, encompassing all “-omics” fields, plays a central role in converging diverse elements of personalized medicine. In the following article, genetic variations in pharmacogenes, their clinical relevance as biomarkers, and the legacy of research in pharmacogenetics are discussed [318]. The growing impact of pharmacogenomics is demonstrated by FDA approvals of personalized therapeutics involving biomarkers. Personalized medicine considerations include genetic factors affecting flavonoid metabolism and response. Pharmacogenomic approaches can help identify patients most likely to benefit from flavonoid therapy. Combination with lifestyle interventions, including diet modification and exercise, can enhance the therapeutic effects of flavonoids. These integrated approaches address multiple aspects of inflammatory disease management.

#### 4.5.4. Regulatory Considerations

Natural product development challenges include standardization of flavonoid content, identification of active compounds, and establishment of quality control measures. Regulatory agencies require extensive documentation of safety and efficacy for therapeutic purposes.

Safety assessment requirements include comprehensive toxicological studies, drug interaction potential evaluation, and long-term safety monitoring. While flavonoids generally show excellent safety profiles, rigorous evaluation is required for therapeutic applications.

Clinical trial design considerations include appropriate patient selection, outcome measures, and study duration. The design of clinical trials for flavonoids requires consideration of their unique pharmacological properties.

Intellectual property issues surrounding natural products and their derivatives affect commercial development prospects. Patent strategies for flavonoid-based therapeutics must consider both composition and method-of-use claims, but it is nearly impossible to secure patents on natural molecules.

## 5. Challenges and Future Directions

### 5.1. Current Limitations

Bioavailability and pharmacokinetic challenges remain significant barriers to flavonoid therapeutic development. The poor absorption, extensive metabolism, and rapid elimination of most flavonoids limit their therapeutic potential. This raises serious concerns about whether the low micromolar IC_50_ values observed experimentally are achievable in vivo, particularly at target sites such as the coronary microvasculature. Advanced delivery systems and structural modifications are needed to overcome these limitations.

The following comparative Table 8 summarizes a weighted meta-analysis of flavonoid potency in inhibiting human MC degranulation, expressed as IC_50_ values derived from multiple experimental systems with preference given to human LAD2 and primary MC models. Among the ten compounds evaluated, luteolin and quercetin emerge as the most potent inhibitors with low micromolar IC_50_ values, while flavanones such as naringenin and hesperetin show substantially weaker activity in the high micromolar range. The ranking reflects a clear structure–activity relationship in which planar flavonoids bearing a catechol B-ring and C2=C3 double bond appear to demonstrate superior MC stabilizing activity, though it should be noted that evidence quality varies across compounds, with several IC_50_ estimates derived from rodent or leukemic cell lines rather than primary human MCs.

Individual variability in flavonoid response due to genetic differences, gut microbiota composition, and lifestyle factors complicates therapeutic applications. Personalized approaches based on individual characteristics may be needed for optimal therapeutic outcomes.

Standardization of natural products remains challenging due to variability in plant sources, extraction methods, and analytical techniques. Consistent quality and potency are essential for therapeutic applications.

Flavonoids may also have pro-oxidative effects due to their metal-reducing properties. A study comparing 24 structurally related flavonoids for copper reduction and modulation of the Fenton reaction found that most reduced cupric ions, with flavone being the only one that potentiated copper-triggered hemolysis [331]. Moreover, estrogenic soy flavonoids should be avoided in cases of estrogen-dependent breast and ovarian cancers. In addition, high amounts of flavonoids (>2000 mg/day) will inhibit gut and liver metabolizing enzymes, especially in those with polymorphisms [228,229,332].

A key limitation in the current literature is the heterogeneity and, at times, contradiction in reported effects on MC modulation across experimental systems. While many in vitro and animal studies demonstrate robust inhibition of MC degranulation and inflammatory signaling, these findings are not consistently replicated in human studies. This likely reflects differences in MC phenotypes (e.g., Laboratory of allergic diseases 2 (LAD2) human mast cells vs. primary human cells), variability in stimuli (IgE-dependent vs. neuropeptide-mediated activation), and the use of non-physiological flavonoid concentrations in vitro. Addressing these translational gaps will require a deliberate shift toward physiologically relevant experimental models to better bridge the distance between mechanistic insights and clinical application.

### 5.2. Emerging Technologies

Advanced delivery systems, including targeted nanoparticles, cell-penetrating peptides, and tissue-specific delivery vehicles, offer solutions to bioavailability challenges. These technologies can improve therapeutic indices and reduce side effects.

Recent advances in nanotechnology provide a plausible pathway to bridge the gap between in vitro flavonoid efficacy and clinical application. Lipid-based delivery systems, including liposomes, nanoemulsions, and solid lipid nanoparticles, have demonstrated the ability to enhance flavonoid solubility, protect against metabolic degradation, and improve systemic bioavailability, thereby addressing key pharmacokinetic limitations [230,333]. Importantly, these carriers can facilitate targeted delivery across biological barriers, including the vascular endothelium and potentially the blood–brain barrier, which is particularly relevant for neuroimmune regulation in cardiovascular disease [333]. Emerging next-generation lipid nanoparticles further enable organ-specific delivery and controlled release, although challenges such as drug leakage, limited targeting specificity, and scale-up reproducibility remain barriers to clinical translation [334]. Recent studies on nanoparticle-based flavonoid formulations (e.g., rutin and chrysin) demonstrate improved pharmacokinetics and enhanced therapeutic efficacy in preclinical models, supporting their potential for cardiovascular and inflammatory indications [230]. However, successful clinical implementation will require standardized formulation strategies, rigorous pharmacokinetic validation, and integration of carrier design with disease-specific targeting to ensure that enhanced bioavailability translates into meaningful clinical outcomes.

Precision medicine approaches utilizing pharmacogenomics, biomarker identification, and individualized dosing strategies can optimize flavonoid therapy. Understanding individual variations in response can improve therapeutic outcomes.

Biomarker development for monitoring flavonoid effects and predicting therapeutic responses is an active area of research. Reliable biomarkers can guide dosing decisions and assess treatment efficacy.

### 5.3. Research Gaps and Opportunities

Mechanistic understanding gaps remain in several areas, including tissue-specific effects, long-term consequences of chronic exposure, and interactions with other therapeutic agents. Addressing these gaps will facilitate clinical translation.

Long-term safety evaluation requires extended studies to assess potential cumulative effects and identify rare adverse reactions. While short-term safety is well-established, long-term data are limited for many flavonoids.

Clinical translation challenges include identifying optimal dosing regimens, patient selection criteria, and appropriate outcome measures. Successful translation requires careful consideration of these factors.

### 5.4. Future Therapeutic Applications

Cardiac immunology [179,335], especially cardiac inflammation [167,179,198,200,336], has surfaced as a key risk factor to be addressed in CVD. There is a lack of clinically available drugs that inhibit the activation of MCs.

Novel disease targets for flavonoid therapy continue to emerge as understanding of their mechanisms expands. Neuroinflammatory diseases, metabolic disorders, and age-related conditions represent promising new applications.

Preventive medicine applications leveraging the safety and efficacy of flavonoids could reduce the burden of inflammatory diseases. Early intervention strategies may prevent disease progression and improve long-term outcomes.

Combination therapy development utilizing flavonoids with other natural products or conventional drugs offers opportunities for enhanced therapeutic efficacy. These approaches can address the complexity of inflammatory diseases.

## 6. Conclusions

### 6.1. Summary of Key Findings

This comprehensive review has examined the crucial role of MCs in microvascular inflammation and the therapeutic potential of flavonoids as natural anti-inflammatory agents. MCs, strategically positioned throughout the microvasculature, serve as key initiators and amplifiers of inflammatory responses through their rapid degranulation capabilities and sustained mediator synthesis.

The molecular mechanisms of MC-mediated inflammation involve complex signaling pathways leading to the release of diverse inflammatory mediators, including histamine, proteases, lipid mediators, and cytokines. These mediators collectively promote increased vascular permeability, endothelial dysfunction, and inflammatory cell recruitment, contributing to tissue damage in various pathological conditions.

Flavonoids have emerged as promising therapeutic agents capable of modulating MC function through multiple mechanisms, including degranulation inhibition, mediator synthesis suppression, and antioxidant protection. The extensive body of evidence from in vitro, animal, and clinical studies supports the therapeutic potential of these natural compounds.

### 6.2. Clinical Relevance

The clinical significance of MC-mediated inflammation extends across multiple disease states, including allergic disorders, I/R injury, CVD, and neuroinflammatory conditions. The ability of flavonoids to modulate these inflammatory processes positions them as valuable therapeutic tools for treating diverse pathological conditions.

The relatively favorable safety profiles of flavonoids, combined with their multi-target mechanisms of action, offer advantages over conventional single-target therapies. The potential for combination therapies utilizing flavonoids with conventional drugs provides opportunities for enhanced therapeutic efficacy with reduced side effects.

The growing recognition of inflammation as a common pathological mechanism underlying various chronic diseases highlights the importance of developing effective anti-inflammatory strategies. Flavonoids represent a promising class of compounds for addressing this therapeutic need.

### 6.3. Research Priorities

Critical knowledge gaps remain in understanding the tissue-specific effects of flavonoids, optimal dosing strategies, and long-term safety profiles. Future research should focus on addressing these limitations to facilitate clinical translation.

The development of improved delivery systems and structural modifications to enhance bioavailability represents a high priority for flavonoid therapeutic development. Advanced formulation technologies and personalized medicine approaches offer promising solutions.

Collaborative research efforts involving academic institutions, pharmaceutical companies, and regulatory agencies will be essential for advancing flavonoid-based therapeutics from laboratory discoveries to clinical applications. Such collaborations can accelerate the development process and ensure rigorous evaluation of safety and efficacy.

The integration of flavonoid therapy into clinical practice will require comprehensive education of healthcare providers and development of evidence-based treatment guidelines. This educational component is crucial for the successful implementation of flavonoid-based therapeutic strategies. Table 9 depicts the current status, limitations, and future directions for flavonoid-based MC therapeutics (Objectives 8, 9, and 10).

In conclusion, the therapeutic potential of flavonoids for treating MC-mediated inflammatory conditions is substantial, supported by extensive scientific evidence and favorable safety profiles. Continued research and development efforts focused on overcoming current limitations will likely lead to successful clinical applications of these promising natural anti-inflammatory agents.

## Figures and Tables

**Table 1:** Mast cell biology and microvascular localization.

Theme/Topic	Key Findings	Studies
MC Development and Phenotypic Diversity	MCs originate from hematopoietic lineage with distinct developmental pathways; exhibit phenotypic heterogeneity based on tissue microenvironment; express unique surface markers (c-Kit, FcεRI) for identification and activation	[13,39,40,41,47]
Perivascular MC Localization	MCs strategically positioned adjacent to blood vessels for immune surveillance; utilize integrin β1-mediated mechanisms to probe vessel content; maintain close proximity to microvascular endothelium for rapid response	[12,44,48,49]
Cardiac MC Distribution	Human cardiac tissue contains significant MC populations; MCs localize in atrial and ventricular myocardium; increased MC density observed in pathological cardiac conditions; strategic positioning near coronary microvasculature	[50,51,52,53,54]
MC-Vascular Endothelium Interaction	MCs modulate vascular and lymphatic endothelial function; participate in bidirectional communication with endothelial cells; influence microvascular permeability and tone through mediator release	[6,12,55,56]

**Table 2:** Molecular mechanisms of MC-mediated microvascular inflammation.

Theme/Topic	Key Findings	Studies
FcεRI Signaling and Degranulation	IgE-mediated activation through FcεRI triggers calcium influx and degranulation; distinct degranulation strategies based on activation signals (anaphylactic vs. piecemeal); phosphorylation cascades involving Lyn, Syk kinases; SHIP1-mediated signal regulation	[66,68,116,120,127]
Preformed Mediator Release	Histamine induces coronary vasoconstriction/vasodilation via H1/H2 receptors; tryptase serves as biomarker for MC activation in cardiovascular disease; chymase contributes to angiotensin II formation and tissue remodeling; heparin affects coagulation and inflammation	[72,96,97,101,128,129]
De Novo Lipid Mediator Synthesis	Arachidonic acid metabolism produces leukotrienes (LTC4, LTD4, LTE4) and prostaglandins; PAF synthesis contributes to platelet activation and microthrombosis; eicosanoid production affects vascular tone and permeability; COX-2 and 5-LOX pathways activated	[69,92,107,108,130,131]
Cytokine and Chemokine Production	IL-6, TNF-α, IL-1β production amplifies inflammatory cascades; IL-33 acts as alarmin triggering MC activation; synergistic effects with neuropeptides (substance P, neurotensin); differential cytokine release via c-kit vs. FcεRI pathways	[43,46,67,112,114,132,133]
MAPK and NF-κB Signaling	ERK, JNK, p38 MAPK pathways regulate inflammatory gene expression; NF-κB activation controls transcription of pro-inflammatory mediators; mTOR pathway involved in MC metabolic regulation and mediator synthesis; NFAT pathway regulates cytokine production	[121,123,134,135,136]

**Table 3:** Mast cell mediators affecting heart function.

Mediator	Pathologic Effect
Chymase	Angiotensin II production–vasoconstriction
Epidermal growth factor (EGF)	Kidney function
Histamine	Coronary constriction
IL-1beta	Inflammation
IL-1 soluble receptor	Inflammation
IL-6	Inflammation, MC proliferation
IL-8 (CXCL8)	Leucocyte chemotaxis
IL-18	Chronic inflammation
IL-31	Pruritus
IL-33	Inflammation
IL-33 soluble receptor	Inflammation
Monocyte chemoattractant peotein-1 (MCP-1)	Monocyte and mast cell recruitment
Metalloproteinase-9 (MMP-9)	Inflammation, tissue damage
Osteopontin	Fibrosis
TGFbeta	Fibrosis
TNFalpha	Inflammation
Tryptase	PAR2 activation, inflammation

**Table 4:** Pathophysiological roles of MCs in disease states.

Theme/Topic	Key Findings	Studies
Cardiovascular Disease	MCs accumulate in atherosclerotic plaques and promote lesion progression; tryptase levels correlate with CAD severity; MC activation contributes to plaque instability and rupture; histamine-induced coronary spasm in allergic angina (Kounis syndrome)	[77,142,143,157,158,168,170]
Ischemia–Reperfusion Injury	MC degranulation exacerbates I/R injury in the heart and other organs; MC-deficient mice show reduced cardiac necrosis post-I/R; chymase and tryptase contribute to tissue damage; early MC stabilization provides cardioprotection	[148,149,150,164]
Heart Failure and Cardiac Remodeling	MC density increases in failing myocardium; tryptase levels inversely correlate with left ventricular function; MC-fibroblast interactions promote myocardial fibrosis; chymase contributes to adverse cardiac remodeling	[117,161,165,174,175]
COVID-19 and Viral Myocarditis	SARS-CoV-2 spike protein activates MCs, contributing to inflammation; MC activation implicated in long-COVID cardiovascular complications; increased MC infiltration in COVID-19 myocarditis; microvascular dysfunction linked to MC mediators	[141,191,192,194,198,200]
Neuroinflammatory Conditions	MCs disrupt blood–brain barrier integrity; accumulate in brain pathologies, including Alzheimer’s disease; stress-induced MC activation affects neuronal function; MC mediators contribute to neuroinflammation and cognitive impairment	[22,60,62,63,201]
Autoimmune and Allergic Disorders	MC-T cell interactions amplify autoimmune responses; anaphylaxis causes systemic cardiovascular collapse via MC mediators; mast cell activation syndrome (MCAS) presents with multi-organ symptoms; MC involvement in skin inflammatory diseases	[106,139,140,144,145]

**Table 5:** Flavonoid structure, classification, and bioavailability.

Theme/Topic	Key Findings	Studies
Flavonoid Classification	Six major subclasses: flavones (luteolin, apigenin), flavonols (quercetin, kaempferol, myricetin), flavanones (hesperidin), flavanonols, isoflavones, and anthocyanidins; structural differences in hydroxylation and glycosylation patterns determine bioactivity	[24,25,33,227]
Intestinal Absorption	Glycosides undergo deglycosylation by intestinal enzymes; aglycones better absorbed than glycosides; glucose transporters facilitate uptake; extensive first-pass metabolism limits bioavailability; C-glycosides more stable than O-glycosides	[218,224,225]
Metabolism and Biotransformation	Phase I (CYP450) and Phase II (glucuronidation, sulfation, methylation) metabolism; gut microbiota converts flavonoids to bioactive metabolites; metabolites may retain or enhance anti-inflammatory activity; colonic metabolites interact with CYP2D6	[226,228,229]
Pharmacokinetic Limitations	Low oral bioavailability (<10% for most); rapid metabolism and elimination; poor water solubility limits absorption; extensive plasma protein binding; advanced formulation strategies needed to enhance delivery	[224,230]
Structure–Activity Relationships	Hydroxyl group positions affect anti-inflammatory potency; catechol structure in B-ring enhances antioxidant activity; methoxylation can improve stability and cell permeability; glycosylation affects bioavailability but may reduce direct activity	[26,227,231]

**Table 7:** Anti-inflammatory mechanisms of flavonoids on mast cells.

Theme/Topic	Key Findings	Studies
Degranulation Inhibition	Luteolin and quercetin more potent than cromolyn in blocking histamine release; inhibition of FcεRI and MRGPRX2-mediated degranulation; stabilization of mast cell membranes; prevention of calcium influx required for exocytosis	[238,239,240,279,280]
Calcium Signaling Modulation	Flavonoids regulate intracellular Ca^2+^ levels preventing degranulation; inhibition of calcium-dependent protein kinases; modulation of Ca^2+^-ATPase activity; prevention of calcium-triggered exocytosis	[65,278,279,280]
MAPK Pathway Inhibition	Suppression of ERK, JNK, p38 phosphorylation; reduced AP-1 activation; decreased inflammatory gene transcription; luteolin inhibits JNK in IL-6 production; kaempferol modulates MAPK in neuroinflammation	[241,264,282,293]
NF-κB Pathway Suppression	Prevention of NF-κB nuclear translocation; inhibition of IκB degradation; reduced transcription of pro-inflammatory genes (TNF-α, IL-6, IL-1β); synergistic effects with other anti-inflammatory pathways	[234,282,283,293]
mTOR Pathway Modulation	Methoxyluteolin inhibits mTOR activation in human mast cells; suppression of neurotensin-stimulated mediator release; reduced substance P effects via mTOR inhibition; potential in neuroinflammation control	[122,242,294]
Cytokine Synthesis Inhibition	Reduced IL-6, IL-8, TNF-α, IL-1β production; inhibition of IL-33-induced IL-31 secretion; suppression of synergistic cytokine release (SP + IL-33); blocking of de novo inflammatory mediator synthesis	[112,114,241,266,293]
Antioxidant Protection	Direct ROS scavenging through hydroxyl groups; upregulation of Nrf2/HO-1 pathway; enhancement of endogenous antioxidant defenses; protection against oxidative stress-induced MC activation	[28,232,233,264,297]
Lipid Mediator Inhibition	COX-2 and 5-LOX dual inhibition reducing prostaglandins and leukotrienes; PAF synthesis suppression; modulation of arachidonic acid metabolism; phospholipase A2 inhibition	[235,252,254,274]

**Table 8:** Weighted IC_50_ values of flavonoids inhibiting human mast cell degranulation.

Rank	Flavonoid	Subclass	Weighted IC_50_ (µM)	Experimental Model(s)	Degranulation Readout	Key Reference(s)
1	Luteolin	Flavone	~1–10 µM (range across stimuli)	Human Cultured Mast Cells (HCMCs), LAD2	Histamine, Leukotrienes (LT), prostaglandin D2 (PGD2), Granulocyte-macrophage colony-stimulating factor (GM-CSF)	[280,283,319]
2	Quercetin	Flavonol	~5–10 µM	HCMCs, human cord blood-derived mast cells (hCBMCs), basophils	Histamine, IL-6, IL-8, TNF-α	[26,237,280,319]
3	Apigenin	Flavone	<10 µM (RBL-2H3); human primary data limited	RBL-2H3, HMC-1	β-hexosaminidase	[273,320]
4	Fisetin	Flavonol	~13.6 µM (RBL-2H3); 25–50 µM in LAD2	RBL-2H3, LAD2, HMC-1	β-hexosaminidase, cytokines	[281,321]
5	Kaempferol	Flavonol	~10–25 µM (RBL-2H3); unclear in primary human	RBL-2H3, HMC-1, LAD2	Histamine, β-hexosaminidase	[264,312,322]
6	Myricetin	Flavonol	~15–25 µM (estimated)	Rat Basophilic Leukemia cells (RBL-2H3), HMC-1	Histamine, TNF-α, IL-6	[263,265,322]
7	Baicalein	Flavone	~20–30 µM	HCMCs	Histamine, LT	[319,323]
8	Epigallocatechin gallate	Flavan-3-ol	~30–50 µM (estimated)	Rat peritoneal MCs, HMC-1	Histamine, β-hexosaminidase	[324,325]
9	Naringenin	Flavanone	~50–100 µM	RBL-2H3, basophils	Histamine	[326,327]
10	Hesperetin	Flavanone	~80–120 µM	RBL-2H3, basophils	β-hexosaminidase, histamine	[328,329,330]

**Table 9:** Clinical translation and therapeutic development challenges.

Theme/Topic	Key Findings	Studies
Clinical Evidence	Randomized controlled trials show efficacy in allergic conditions; open-label studies demonstrate benefits in autism and neuroinflammation; successful COVID-19 case reports using integrated MC-targeting approach; meta-analyses support cardiovascular and metabolic benefits	[20,27,269,271,306]
Bioavailability Enhancement	Nanoparticle formulations improve delivery and bioavailability; metal-polyphenol complexes enhance stability; sustained-release systems extend therapeutic window; topical formulations for localized effects	[230,251,310,311,312]
Structure–Activity Optimization	Methoxylation improves potency and stability (methoxyluteolin); hydroxyl group positioning critical for activity; glycosylation affects pharmacokinetics; need for systematic SAR studies	[227,231,240]
Safety and Tolerability	Generally well-tolerated with wide safety margins; few adverse effects reported in clinical trials; potential drug interactions via CYP450 inhibition; need for long-term safety data	[229,251,308,332]
Pharmacogenomics Considerations	Genetic polymorphisms in metabolizing enzymes affect response; potential for personalized flavonoid therapy; need for pharmacogenomic studies	[318]
Standardization and Quality Control	Variable flavonoid content in natural products; importance of standardized extracts; need for validated analytical methods; regulatory considerations for supplements vs. drugs	[314]
Mechanistic Gaps	Need for more detailed molecular target identification; clarification of metabolite contributions to activity; understanding tissue-specific effects; elucidation of multi-target synergistic mechanisms	[226,227]
Future Research Priorities	Large-scale RCTs for specific inflammatory conditions; development of optimized synthetic derivatives; combination therapy approaches; biomarker-guided therapy; precision medicine applications targeting MC-mediated diseases	[57,140]

## Data Availability

No new data were created or analyzed in this study.
